# Synthesis of Neoglycoconjugates Containing 4-Amino-4-deoxy-l-arabinose Epitopes Corresponding to the Inner Core of *Burkholderia* and *Proteus* Lipopolysaccharides

**DOI:** 10.1002/ejoc.201101171

**Published:** 2011-11-16

**Authors:** Markus Blaukopf, Bernhard Müller, Andreas Hofinger, Paul Kosma

**Affiliations:** 1 Department of Chemistry, University of Natural Resources and Life SciencesMuthgasse 18, 1190 Vienna, Austria

**Keywords:** Biomimetic synthesis, Glycoconjugates, Glycosylation, Glycolipids, Lipopolysaccharide, Carbohydrates

## Abstract

Disaccharides that contain 3-deoxy-d-*manno*-oct-2-ulosonic acid (Kdo) and d-*glycero*-d-*talo*-oct-2-ulosonic acid (Ko) substituted at the 8-position by 4-amino-4-deoxy-β-l-arabinopyranosyl (Ara4N) residues have been prepared. Coupling an *N*-phenyltrifluoroacetimidate-4-azido-4-deoxy-l-arabinosylglycosyl donor to acetyl-protected allyl glycosides of Kdo and Ko afforded anomeric mixtures of disaccharide products in 74 and 90 % yield, respectively, which were separated by chromatography. Further extension of an intermediate Ara4N-(1→8)-Kdo disaccharide acceptor, which capitalized on a regioselective glycosylation with a Kdo bromide donor under Helferich conditions, afforded the branched trisaccharide α-Kdo-(2→4)[β-l-Ara4N-(1→8)]-α-Kdo derivative. Deprotection of the protected di- and trisaccharide allyl glycosides was accomplished by TiCl_4_-promoted benzyl ether cleavage followed by the removal of ester groups and reduction of the azido group with thiol or Staudinger reagents, respectively. The reaction of the anomeric allyl group with 1,3-propanedithiol under radical conditions afforded the thioether-bridged spacer glycosides, which were efficiently coupled to maleimide-activated bovine serum albumin. The neoglycoconjugates serve as immunoreagents with specificity for inner core epitopes of *Burkholderia* and *Proteus* lipopolysaccharides.

## Introduction

4-Amino-4-deoxy-l-arabinose (Ara4N) constitutes an important sugar component within the outer membrane of Gram-negative bacteria. Thus, Ara4N residues occur in substoichiometric to stoichiometric amounts ester-linked to the 1- and 4′-phosphate groups of the glucosamine disaccharide backbone of lipid A, which anchors the lipopolysaccharide (LPS) chain to the outer membrane.^1^–^3^ In addition, Ara4N residues have also been detected glycosidically-linked to the LPS core sugar 3-deoxy-d-*manno*-oct-2-ulosonic acid (Kdo), which provides the linkage unit to the lipid A domain. In particular, Ara4N has been found in a β-(1→8) linkage to Kdo in the inner core region of many *Proteus* strains and in a *Providencia* strain.^4^–^6^ Bacteria of the genus *Proteus* are important pathogens that cause nosocomial wound infections and urinary tract infections. Similarly, Ara4N has been detected linked to the 8-position of the Kdo-isosteric sugar d-*glycero*-d-*talo*-oct-2-ulosonic acid (Ko) in the core region of *Burkholderia*^7^–^10^ and in a *Serratia marcescens* strain.^11^ *Burkholderia* species are responsible for severe and fatal infections in cystic fibrosis sufferers. The incorporation of Ara4N in the lipid A and core regions has been implicated in the development of antibiotic resistance in bacteria by masking the anionic charges of the phosphate and carboxylic acid groups, which counteracts the effects of cationic antimicrobial peptides.^12^ Previously, antibodies raised against neoglycoconjugates containing the Ko-(2→4)-Kdo disaccharide did not bind to the LPS of *Burkholderia*, which could be because of steric hindrance by the terminal Ara4N residue, which masks Ko and Kdo epitopes.^13^ In order to study the antigenic properties of Ara4N-substituted inner core LPS units and develop poly- and monoclonal antibodies for diagnostic applications, we have set out to synthesize the central Ara4N-Kdo/Ko units that correspond to part of the structures of the *Burkholderia* and *Proteus* LPS. Herein we present results pertaining to the development of new Ara4N glycosyl donors, assembly of the di- and trisaccharide units, and conversion into the corresponding neoglycoconjugates. An additional feature relates to the orthogonal utilization of allyl and benzyl protecting groups, which considerably adds to the repertoire of protecting group strategies in oligosaccharide syntheses.

## Results and Discussion

**Scheme 1:**
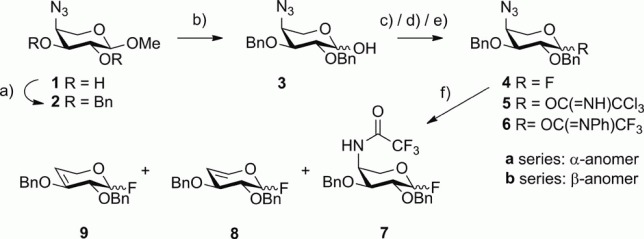
Reagents and conditions for the synthesis of Ara4N glycosyl donors. (a) BnBr, NaH, DMF, room temp., 1 h, 93 %; (b) AcOH, 2 M HCl, 65 °C, 15 h, 61 %; (c) DAST, CH_2_Cl_2_, –20 °C, 2 h, 99 %; (d) Cl_3_CCN, K_2_CO_3_, CH_2_Cl_2_, room temp., 12 h, 78 %;(e) F_3_C(Cl)C=NPh, K_2_CO_3_, CH_2_Cl_2_, room temp., 12 h, 84 %; (f) (Ph)_3_P, (F_3_CCO)_2_O, CH_2_Cl_2_, room temp., 1 h, 47 % for **7**.

Multigram amounts of methyl 4-azido-4-deoxy-α-l-arabinopyranoside (**1**) have been generated from commercial methyl β-d-xylopyranoside.^14^ Benzyl groups were selected as nonparticipating protecting groups at the 2- and 3-positions, and **1** was converted into the 2,3-di-*O*-benzyl glycoside **2** by reaction with benzyl bromide/NaH in *N*,*N*-dimethylformamide (DMF) in 93 % yield (Scheme 1). In order to generate hemiacetal **3**, which was the precursor for the armed glycosyl donors **4**–**7**, hydrolysis of **2** was accomplished by treatment with 2 M HCl in acetic acid at 65 °C, and lactol **3** was isolated after flash chromatography in 61 % yield. As fluoride glycosyl donors have served as efficient donors in numerous glycoside syntheses,^15^ including coupling to 8-*O*-silylated Kdo derivatives,^16^ donor **4** was prepared by the reaction of **3** with diethylaminosulfur trifluoride (DAST) in CH_2_Cl_2_ at –20 °C to afford an anomeric mixture of 4-azido-4-deoxy-l-arabinopyranosyl fluoride (**4**) in ca. 99 % yield with preferential formation of the equatorial isomer (α/β ratio ca. 2:1). In addition, the trichloroacetimidate donor **5** (α/β ratio ca. 3:1) was prepared from the reaction of **3** with trichloroacetonitrile/K_2_CO_3_ at room temperature in 78 % yield.^17^ Similarly, conversion of **3** into the *N*-phenyltrifluoroacetimidate derivative **6** (α/β ratio ca. 7:1) was effected by treatment of **3** with *N*-phenyltrifluoroacetimidoyl chloride/K_2_CO_3_ in acetone in 84 % yield.^18^ In order to exploit the stabilization of a putative oxacarbenium ion intermediate in the glycosylation step by the intramolecular participation of a 4-acylamino moiety, the 4-deoxy-4-trifluoroacetamido derivative **7** was also prepared.^19^,^20^ The azido group of **4** was subjected to a Staudinger reaction with triphenylphosphane and trifluoroacetic anhydride in CH_2_Cl_2_, which gave a moderate yield (47 %) of the corresponding 4-deoxy-4-trifluoroacetamidoarabinosyl fluoride derivative **7** together with the byproducts **8** and **9** (35 % combined yield), which were removed by column chromatography. The anomeric mixtures of **5**, **6**, and **7** were resolved by silica gel chromatography.

**Scheme 2:**
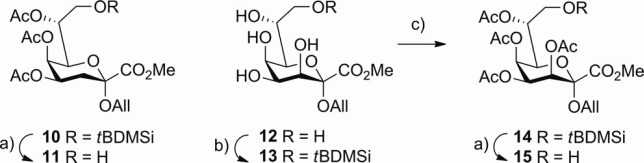
Reagents and conditions for the synthesis of Kdo and Ko glycosyl acceptors. (a) 2 % HF, MeCN, room temp., 2 h, ca. quant.; (b) *t*BDMSCl, DABCO, MeCN, room temp., 15 h, then (c) Ac_2_O, DMAP, pyridine, room temp., 12 h, 62 %.

For the straightforward assembly of the (1→8)-linked disaccharide units, the peracetylated 8-*O*-*tert*-butyldimethylsilyl allyl glycoside derivatives of Kdo (**10**) and Ko (**14**) were chosen as glycosyl acceptors that allow direct coupling with fluoride donors and provide access to the respective alcohol by reaction with fluoride ions. Although the Kdo derivative **11** had previously been used for the preparation of the *Chlamydia*-related 2→8 interlinked Kdo disaccharide,^21^ regioselective silylation of the known^22^,^23^ Ko allyl glycoside methyl ester derivative **12** had to be established (Scheme 2). Gratifyingly, the reaction of **12** with *tert*-butyldimethylsilyl chloride (*t*BDMSCl) in dry acetonitrile in the presence of diazabicyclo[2.2.2]octane (DABCO) afforded the 8-*O*-silyl derivative **13**, which was acetylated with acetic anhydride/4-(dimethylamino)pyridine (DMAP) in pyridine to furnish the 3,4,5,7-tetra-*O*-acetyl Ko glycosyl acceptor **14** in 62 % overall yield. The structural assignment of **14** was based on the low-field chemical shifts observed for protons H-3, H-4, H-5, and H-7 in the ^1^H NMR spectrum. Removal of the 8-*O*-*t*BDMS groups from **10** and **14** was effected by treatment with 2 % HF in acetonitrile to give the alcohols **11** and **15**, respectively, in near quantitative yields. As the 7-*O*-acetyl group is prone to migration to the 8-position upon contact with silica gel, the crude products of **11** and **15** were used for the subsequent glycosylation step.

**Scheme 3:**
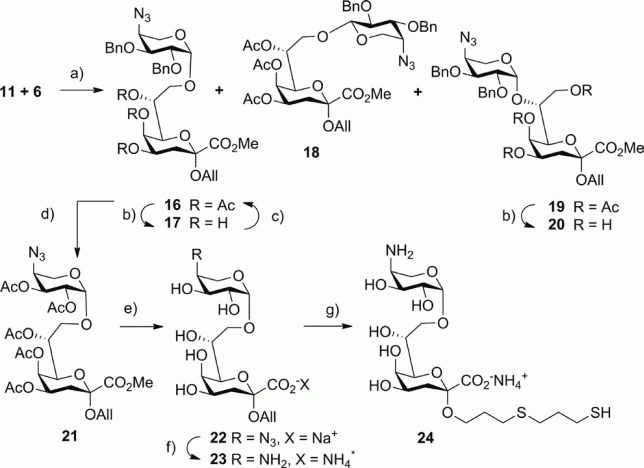
Reagents and conditions for the synthesis of Ara4N-(1→8)-Kdo disaccharide derivatives. (a) TMSOTf (0.05 equiv.), CH_2_Cl_2_, –25 °C, 1 h, 74 %; (b) 0.1 M NaOMe, MeOH, room temp., 4 h, 30 % for **20**, 69 % for **17**; (c) Ac_2_O, DMAP, pyridine, room temp., 24 h, 80 %; (d) TiCl_4_, CHCl_3_, 0 °C, 12 h, then Ac_2_O, DMAP, pyridine, room temp., 24 h, 68 %; (e) 0.1 M NaOMe, MeOH, then 0.2 M NaOH, H_2_O, room temp., 3 h, 98 %; (f) dithiothreitol, aq. diisopropylamine, room temp., 2 h; then Dowex H^+^, 1 M aq. NH_3_, 60 %; (g) HS(CH_2_)_3_SH, H_2_O, *hν*, room temp., 1 h, BioGel P-2, 63 %.

The glycosylation conditions were elaborated with the Kdo acceptor derivatives **10** and **11**. Coupling reactions of **10** and **4** in the presence of 1.1 equiv. of BF_3_**·**Et_2_O as the promoter in CH_2_Cl_2_ and acetonitrile, respectively, afforded low yields of glycosides (Table 1, Entries 1 and 2). Employing a large excess of promoter provided good yields of the glycosides with a very moderate stereoselectivity in favor of the *cis*-configured β-l-Ara4N glycosides (Entries 3 and 4). The trimethylsilyl triflate (TMSOTf)-promoted coupling reaction of **7** with **15** did not improve the stereochemical outcome of the reaction and gave a 55 % yield of disaccharide (Entry 5). Coupling of **5** with **11** also gave a modest yield of products (Entry 6), whereas less reactive, armed **6** led to good yields of Ara4N-Kdo and Ara4N-Ko glycosides albeit with no improvement in anomeric selectivity (Entry 7).^24^ The disaccharide mixture generated from the reaction of **11** with **6** was partially resolved by chromatography, which allowed the separation of the equatorially-linked α-l-glycoside **18** (Scheme 3). The assignment of the anomeric configuration of the Ara4N residue in **18** was based on the value of the coupling constant *J*_1′,2′_ of ca. 6.2 Hz, which indicated a 1,2-*trans*-glycosidic linkage. The attachment site was identified by the low-field-shifted C-8 Kdo signal at 67.35 ppm in the ^13^C NMR spectrum.

**Table 1:** Glycosylation conditions and product yields.^[a]^

Entry	Donor	Acceptor	Promotor	Solvent	Yield [%]	ax/eq ratio[b]
1	**4**	**10**	BF_3_**·**Et_2_O	CH_2_Cl_2_	17	2:1
2	**4**	**10**	BF_3_**·**Et_2_O	MeCN	21	1:2
3	**4**	**10**	BF_3_**·**Et_2_O[c]	CH_2_Cl_2_	71	1.4:1
4	**4**	**14**	BF_3_**·**Et_2_O[c]	Et_2_O	80	1.5:1
5	**7**	**15**	TMSOTf	CH_2_Cl_2_	55	1.2:1
6	**5**	**11**	TMSOTf	CH_2_Cl_2_	38	1.2:1
7	**6**	**11**	TMSOTf	CH_2_Cl_2_	74	1.6:1
8	**6**	**15**	TMSOTf	CH_2_Cl_2_[d]	90	1.6:1

[a] Reactions were performed at –25 °C.

[b] From integration of the anomeric ^1^H NMR signals.

[c] 10 equiv. of promoter.

[d] Performed at –78 °C.

Subsequently, the 7-substituted byproduct **19** ― arising from acetyl migration to the 8-position in **11** ― was removed by Zemplén deacetylation of the mixture of **16** and **19** to give **17** and **20** in 69 and 30 % yield, respectively. Based on the amount of **11**, the overall yield of desilylation, glycosylation, and isolation of **17** was 36 %. The glycosylation sites were assigned from the chemical shifts of C-8 in the ^13^C NMR spectrum of **17** (70.64 ppm) and C-7 in that of **20** (81.87 ppm). In addition, a diagnostic HMBC correlation from the anomeric proton of the Ara4N residue to C-7 of the Kdo unit was observed in **20**. The *cis* configuration of the arabinopyranosyl units was corroborated by the small value of the coupling constant *J*_1′,2′_ of ca. 3.6 Hz. The triol derivative **17** was reacetylated to afford pure **16** in 80 % yield. Deprotection of **16** was accomplished by a Lewis acid-promoted cleavage of the 2′-*O*- and 3′-*O*-benzyl groups in the presence of TiCl_4_.^25^ This transformation was accompanied by minor hydrolysis of the glycosidic linkages. The intermediate 2′,3′-diol was subsequently acetylated to afford the penta-*O*-acetyl derivative **21** in 68 % overall yield. The ^1^H NMR spectroscopic data for **21** compare favorably with those reported for a related 4′-acetamido-4′-deoxy derivative, which was prepared by chemical transformation of a disaccharide isolated from the *Proteus mirabilis* LPS.4a Prior to reduction of the 4-azido group of **21**, the acetyl groups were removed by transesterification in methanol in order to prevent formation of a 4′-acetamido product. Thus, **21** was subjected to Zemplén deacetylation followed by hydrolysis of the Kdo methyl ester group to furnish the 4′-azido-4′-deoxy-disaccharide derivative **22** in 95 % yield, which was purified by chromatography on BioGel P2. The reduction of the azido group of **22** was accomplished by treatment with dithiothreitol (DTT)^26^ in aqueous pyridine/diisopropylamine, which left the allyl group intact and provided the 4′-amino-4′-deoxy-l-arabinopyranosyl derivative **23**. Purification was performed by ion-exchange chromatography on Dowex WX50 (H^+^ form) eluted with 1 M aqueous NH_3_ followed by desalting on BioGel P2 to give the β-l-Ara4N-(1→8)-Kdo allyl glycoside as the ammonium salt **23** in 60 % yield.

In order to produce neoglycoconjugates with covalently-linked Ara4N-K(d)o ligands, suitable spacer groups were introduced by using the remaining allylic function. The conjugation chemistry has to be compatible with the presence of both amino and carboxylic acid groups in order to retain the antigenic properties of these LPS inner core sugars. Hence, frequently-employed chain-elongation methods by addition of cysteamine or ω-mercaptoalkanoic acid derivatives to the allyl aglycon were not feasible.^27^,^28^ Conjugation conditions also have to take into account the acid-labile glycosidic linkages of the Kdo residues. Hence, thiol-based conjugation chemistry was envisaged to provide chemoselective ligation to the protein matrix.^29^ The introduction of the thiol-based spacer moiety was effected by UV-mediated addition of 1,3-propanedithiol to the allylic aglycon to give the thioether-bridged thiol **24** in 63 % yield.^14^ The resulting material contained a small fraction of the corresponding dimer and was immediately used for the preparation of the glycoconjugate.

**Scheme 4:**
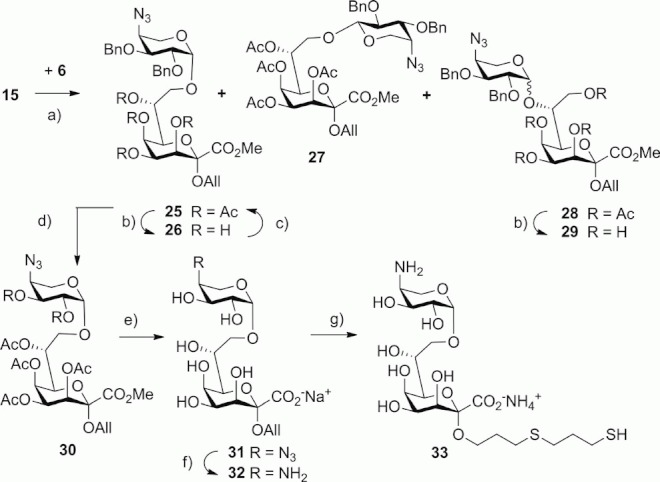
Reagents and conditions for the synthesis of Ara4N-(1→8)-Ko disaccharide derivatives. (a) see Table 1; (b) 0.1 M NaOMe, MeOH, room temp., 4 h 68 % for **26**, 30 % for **29**; (c) Ac_2_O, DMAP, pyridine, room temp., 24 h, 94 %; (d) TiCl_4_, CHCl_3_, 0 °C, 6 h, then Ac_2_O, DMAP, pyridine, room temp., 24 h, 89 %; (e) 0.1 M NaOMe, MeOH, room temp., 4 h, then 0.2 M NaOH, 3 h, 97 %; (f) dithiothreitol, *i*Pr_2_N/H_2_O, room temp., 12 h, then DOWEX (H^+^), 1 M aq. NH_3_, 69 %; (g) HS(CH_2_)_3_SH, H_2_O, *hν*, room temp., 1 h, BioGel P-2, 80 %.

The corresponding Ara4N-Ko glycoside was prepared in a similar way using the conditions established for the Ara4N-Kdo derivatives (Scheme 4). Thus, glycosylation of **15** with **6** proved to be highly efficient and gave a 90 % yield of disaccharide products (Table 1, Entry 8). Again, a minor amount of the α-linked product **27** was formed, which was separated from the mixture of **25** and **28** by chromatography. Final purification of the target disaccharide **25** was achieved by deacetylation with NaOMe, which produced a separable mixture of the triol derivatives **26** (68 %) and **29** (30 %). The overall yield for the preparation of **26** from **15** was 38 %. Subsequent reacetylation of the β-(1→8)-linked disaccharide **26** afforded **25** in 94 % yield. Similar to the Ara4N-Kdo intermediate **16**, the benzyl groups of **25** were cleaved by the action of TiCl_4_ followed by acetylation to furnish the hexa-*O*-acetyl disaccharide **30** in 89 % yield. Zemplén deacetylation and hydrolysis of the Ko methyl ester group gave **31** in 97 % yield. The 4′-azido group of **31** was reduced with dithiothreitol to produce the 4′-amino glycoside **32** in 69 % yield, which was transformed into the thio-spacer derivative **33** in 80 % yield.

**Scheme 5:**
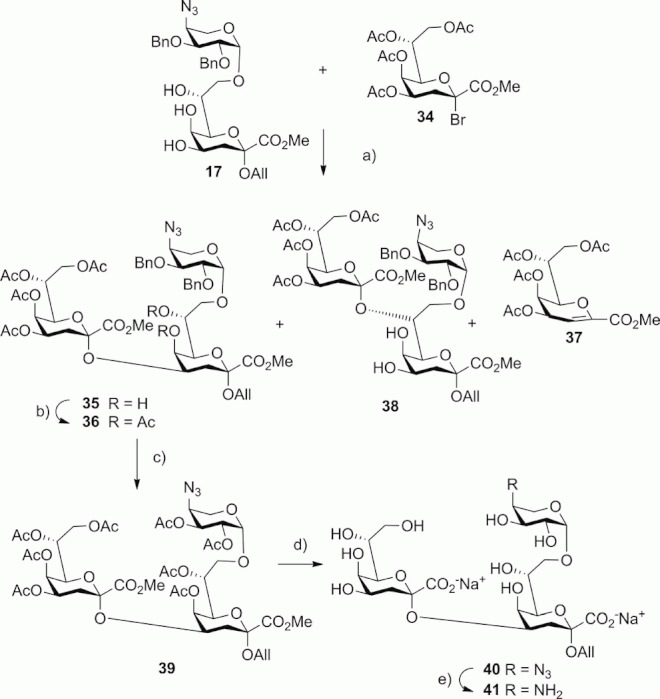
Reagents and conditions for the synthesis of Ara4N-(1→8)-[Kdo-(2→4)]Kdo trisaccharide derivatives. (a) Hg(CN)_2_/HgBr_2_, MeNO_2_, room temp., 14 h, 19 % for **35**, 5.5 % for **38**; (b) Ac_2_O, DMAP, pyridine, room temp., 12 h, 75 %; c) TiCl_4_, CHCl_3_, room temp., 13 h, then Ac_2_O, DMAP, pyridine, room temp., 24 h, 68 %; d) 0.1 M NaOMe, MeOH, room temp., 4 h, then 0.1 M aq. NaOH, room temp., 3 h 96 %; (e) Me_3_P, THF/0.1 M NaOH, room temp., 4 h, 99 %.

Proceeding towards the branched inner core unit related to the *Proteus mirabilis* LPS, the Ara-(1→8)-Kdo intermediate **17** was subjected to a regioselective glycosylation with the known Kdo bromide donor **34**.^30^ The outcome of the Helferich glycosylation procedure was dependant on the solvent system. With CH_2_Cl_2_, the reaction gave a low yield and prevailing formation of the (1→7)-linked product, whereas nitromethane afforded a better yield, regio-, and anomeric selectivity. The excess amount of **34** was limited to avoid formation of the (2→7)-linked trisaccharide **38** and tetrasaccharide byproducts. Separation of **38** and the glycal ester byproduct **37** was achieved by column chromatography followed by HPLC (Scheme 5). Acetylation of **35** afforded the hexa-*O*-acetyl derivative **36** in 75 % yield. A small amount of the β-(2→4)-linked trisaccharide isomer was visible in the ^1^H NMR spectrum of **36**, which was removed in the next step. Deprotection was performed by TiCl_4_-promoted hydrolysis of the benzyl protecting groups followed by acetylation to afford the branched octa-*O*-acetyl trisaccharide derivative **39** in 68 % yield. Remarkably, both of the acid-labile Kdo units were stable during the Lewis acid treatment, and only minor amounts of the hydrolysis products were observed. The ester groups were unblocked by transesterification with NaOMe followed by saponification with aqueous NaOH to give the branched trisaccharide **40**, which was isolated as the disodium salt in 96 % yield. The assignment of the structure of **40** was based on the ^13^C NMR spectrum, which showed low-field-shifted signals for C-4 (69.23 ppm) and C-8 (70.51 ppm) accompanied by high-field-shifted signals of the adjacent carbon atoms (C-7 68.52, C-3 33.94, C-5 64.92 ppm). Reduction of the azido group by treatment with dithiothreitol finally produced the 4″-amino derivative **41** albeit with the formation of a minor byproduct, which was not fully removed by chromatography. Hence, reduction of the azido group was accomplished by treatment of **40** with trimethylphosphane to afford the 4″-amino derivative **41** in 99 % yield.

NMR spectroscopic data for the allyl glycosides **23**, **32**, and **41** are in full agreement with data measured for LPS–core oligosaccharides.^8^

### Conjugation

**Scheme 6:**
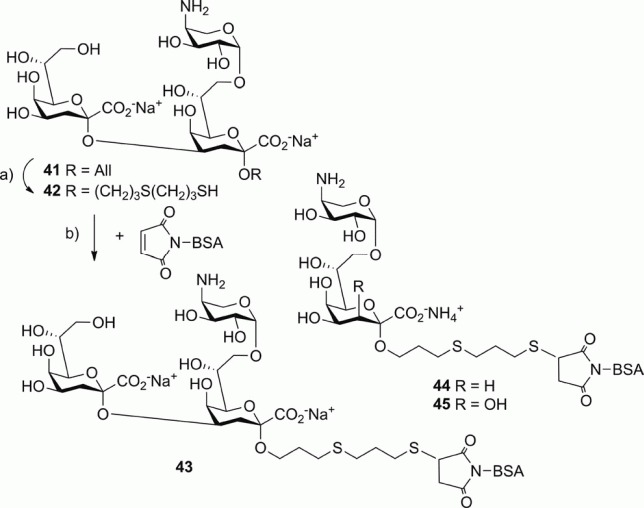
Reagents and conditions for the synthesis of neoglycoconjugates **43**–**45**. (a) HS(CH_2_)_3_SH, H_2_O, *hν*, NH_3_, room temp., 1 h 99 %; (b) room temp., 2 h.

Chain elongation of trisaccharide **41** with 1,3-propanedithiol produced the spacer derivative **42** in excellent yield (Scheme 6). Conjugation of the ligands to maleimide-activated bovine serum albumin (BSA) was achieved by incubation with the thiol spacer-equipped derivatives **24**, **33**, and **42** to furnish the neoglycoconjugates **43**–**45**, which were purified by chromatography on BioGel P2.^31^ The high ligand-to-protein ratios were based on MALDI-TOF analysis (5.4 mol/mol for **43**, 16.5 mol/mol for **44**, and 13.6 mol/mol for **45**). Immunochemical results with polyclonal human and murine sera will be published elsewhere.

## Conclusions

The synthesis of neoglycoconjugates that contain inner core LPS epitopes composed of 4-amino-4-deoxy-l-arabinose residues glycosidically-linked to Kdo and Ko has been accomplished by a straightforward strategy that used a benzylated *N*-phenyl-trifluoroacetimidate Ara4N donor and acetylated allyl glycoside acceptors. Selective cleavage of the benzyl protecting groups in the presence of the allylic aglycon with TiCl_4_ allows the subsequent deprotection of the anomeric center or transformation of the remaining terminal alkene for spacer elongation by Michael addition of the thiol moieties. The spacer ligands are useful probes for the preparation of glyconanoparticles and glycoarrays, and the neoglycoconjugates serve as specific immunoreagents.

## Experimental Section

**General:** Maleimide-activated BSA was purchased from Sigma Aldrich. Known compounds were identified by comparison with reported melting points as well as ^1^H and ^13^C NMR spectroscopic data. Melting points were determined with a Kofler hot stage microscope. Optical rotations were measured with a Perkin–Elmer 243 B Polarimeter. [*α*]_D_^20^ values are given in units of 10^–1^deg cm^2^ g^–1^. ^1^H NMR spectra were recorded at 297 K with a Bruker DPX instrument operating at 300, 400, or 600 MHz for ^1^H with CDCl_3_ as the solvent and Me_4_Si as the standard, unless stated otherwise. ^13^C NMR spectra were measured at 75.47, 100.62, or 150.9 MHz and referenced to 1,4-dioxane (67.40 ppm). Homo- and heteronuclear 2D NMR spectroscopy was performed with Bruker standard software. MALDI-TOF-MS were recorded in the positive ion mode with sinapic acid as matrix. HPLC–HRMS analysis was carried out with H_2_O/MeCN solutions (concentration 1 mg/L) with an HTC PAL system autosampler (CTC Analytics AG), an Agilent 1100/1200 HPLC with binary pumps, degasser, and column thermostat (Agilent Technologies, Waldbronn, Germany), and Agilent 6210 ESI-TOF mass spectrometer (Agilent Technologies, Palo Alto, U.S.). The mass spectrometer was previously tuned with Agilent tune mix and further reference masses were added to provide a mass accuracy below 2 ppm. Data analysis was performed with Mass Hunter software (Agilent Technologies). TLC was performed with Merck precoated plates (5 × 10 cm, layer thickness 0.25 mm, silica gel 60F_254_); spots were detected by spraying with anisaldehyde/H_2_SO_4_. Silica gel (0.040–0.063 mm) was used for column chromatography. Concentration of solutions was performed at reduced pressure at temperatures < 40 °C. Elemental analyses were performed by Dr. J. Theiner, Mikroanalytisches Laboratorium, Institut für Physikalische Chemie, Universität Wien.

**Methyl 4-Azido-2,3-di-*O*-benzyl-4-deoxy-α-l-arabinopyranoside (2):** Sodium hydride (60 % in oil, 1.06 g, 26.5 mmol) was added to a solution of **1**^14^ (2.01 g, 10.6 mmol) in dry DMF (40 mL) at 0 °C and stirred for 10 min. Benzyl bromide (3.04 mL, 28.6 mmol) in DMF (10 mL) was added over 5 min, and the solution was stirred at room temp. for 1 h. The reaction was quenched by addition of methanol (5 mL). The solution was diluted with CH_2_Cl_2_ (50 mL) and washed with water and brine. The organic phase was dried (MgSO_4_) and concentrated. The residue was dried in vacuo to give crude **2** (3.65 g, 93 %) as a yellowish syrup. [*α*]_D_^20^ = +29.4 (*c* = 0.24, CHCl_3_). ^1^H NMR (300 MHz, CDCl_3_): *δ* = 7.41–7.25 (m, 10 H, Ph), 4.85–4.66 (m, 4 H, CH_2_Ph), 4.22 (d, *J*_1,2_ = 6.75 Hz, 1 H, 1-H), 3.95 (dd, *J*_5a,4_ = 3.3, *J*_5a,5b_ = 12.4 Hz, 1 H; 5a-H), 3.79–3.74 (m, 1 H, 4-H), 3.70–3.61 (m, 2 H, 2-H, 3-H), 3.51 (s, 3 H, OCH_3_), 3.43 (dd, *J*_5b,4_ = 1.8 Hz, 1 H, 5b-H) ppm. ^13^C NMR (CDCl_3_, 100 MHz): *δ* = 138.33, 137.71, 128.42, 128.31, 127.96, 127.85, 127.81 and 127.66 (C-Ar), 104.39 (C-1), 79.36 (C-3), 78.57 (C-2), 74.92 (*C*H_2_Ph), 72.82 (*C*H_2_Ph), 62.98 (C-5), 58.57 (C-4), 56.77 (O*C*H_3_) ppm.

**4-Azido-2,3-di-*O*-benzyl-4-deoxy-l-arabinopyranose (3):** A solution of **2** (29.15 g, 79 mmol) in acetic acid (290 mL) and HCl (73 mL, 2 M) was kept at 65 °C for 15 h. The brownish solution was diluted with toluene (450 mL) and cooled to 0 °C. NaHCO_3_ (125 g) was slowly added at 0 °C until CO_2_ formation had ceased. The organic layer was then washed with saturated aqueous NaHCO_3_ and NaCl solutions, dried (MgSO_4_), and concentrated. The residue was dissolved in n-hexane/EtOAc, silica gel was added, and the solvent was removed in vacuo. The remaining solid was subjected to flash chromatography on silica gel (toluene/EtOAc, 3:1) to afford **3** (17.21 g, 61 %) as a colorless solid. M.p. 62–63 °C. [*α*]_D_^20^ = +49.6 (*c* = 0.28, CHCl_3_). ^1^H NMR for β-anomer (400 MHz, CDCl_3_): *δ* = 7.38–7.27 (m, 10 H, Ph), 5.13 (d, *J*_1,2_ = 3.9 Hz, 1 H, 1-H), 4.77–4.66 (m, 4 H, CH_2_Ph), 3.96 (dd, *J*_5a,4_ = 2.4, *J*_5a,5b_ = 11.8 Hz, 1 H, 5a-H), 3.95 (dd, *J*_3,4_ = 3.7, *J*_3,2_ = 7.8 Hz, 1 H, 3-H), 3.82–3.78 (m, 1 H, 4-H), 3.73 (dd, 1 H, 2-H), 3.69 (dd, *J*_5b,4_ = 4.8 Hz, 1 H, 5b-H) ppm. ^13^C NMR (CDCl_3_, 100 MHz): *δ* = 137.64–136.70 (m, C*q*, Ar), 128.89–128.68 (m, C-Ar), 92.00 (C-1), 76.11 (C-3), 76.02 (C-2), 73.75 (*C*H_2_Ph), 73.04 (*C*H_2_Ph), 60.90 (C-5), 58.39 (C-4) ppm. ^1^H NMR for α-anomer (400 MHz, CDCl_3_): *δ* = 7.38–7.27 (m, 10 H, Ph), 4.81 (d, *J*_1,2_ = 3.9 Hz, 1 H, 1-H), 4.77–4.56 (m, 4 H, CH_2_Ph), 4.02 (dd, *J*_5a,4_ = 7.9, *J*_5a,5b_ = 11.8 Hz, 1 H, 5a-H), 3.84 (dd, *J*_3,4_ = 3.2, *J*_3,2_ = 5.8 Hz, 1 H, 3-H), 3.80–3.75 (m, 1 H, 4-H), 3.61 (dd, *J*_5b,4_ = 3.4 Hz, 1 H, 5b-H), 3.57 (dd, 1 H, 2-H) ppm. ^13^C NMR (CDCl_3_, 100 MHz): *δ* = 137.64–136.70 (m, C*q*, Ph), 128.89–128.68 (m, CH, Ph), 94.40 (C-1), 77.50 (C-3), 75.79 (C-2), 73.87 (*C*H_2_Ph), 73.42 (*C*H_2_Ph), 59.04 (C-5), 56.05 (C-4) ppm. C_19_H_21_N_3_O_4_ (355.40): calcd. C 64.21, H 5.96, N 11.82; found C 64.22, H 5.79, N 11.66.

**4-Azido-2,3-di-*O*-benzyl-4-deoxy-l-arabinopyranosyl Fluoride (4):** Diethylaminosulfur trifluoride (111 μL, 0.84 mmol) was slowly added to a solution of **3** (250 mg, 0.70 mmol) in dry CH_2_Cl_2_ (5 mL) at –20 °C. After stirring for 2 h at –20 °C, MeOH (0.5 mL) was added and stirring was continued for a further 10 min. The solution was diluted with CH_2_Cl_2_ (50 mL), washed with saturated aqueous NaHCO_3_, dried (MgSO_4_), and concentrated. The crude yellow oil was purified by column chromatography on silica gel (hexane/EtOAc, 10:1) to give an anomeric mixture of **4** (250 mg, 99 %) as a colorless liquid. ^1^H NMR (600 MHz, CDCl_3_): *δ* = 7.43–7.27 (m, 20 H, 4 × Ph), 5.55 (dd, *J*_1β,F_ = ca. 53.1, *J*_1β,2β_ = ca. 2.6 Hz, 1 H, 1β-H), 5.35 (dd, *J*_1α,F_ = ca. 50.4, *J*_1α,2α_ = 2.6 Hz, 1 H, 1α-H), 4.89–4.56 (m, 8 H, 4 × OC*H*_2_), 4.23–4.18 (m, 1 H, 5aα-H), 4.06 (dd, *J*_3,2_ = 9.7, *J*_3,4_ = 3.7, 1 H, 3β-H), 3.96–3.92 (m, 2 H, 4β-H, 5aβ-H), 3.89 (ddd, *J*_2,F_ = 25.0 Hz, 2β-H), 3.85–3.82 (m, 1 H, 3α-H), 3.97–3.67 (m, 4 H, 2α-H, 4α-H, 5bα-H, 5bβ-H) ppm. ^13^C NMR (150 MHz, CDCl_3_): *δ* = 137.78, 137.65, 137.30, 137.07 (4 × C-Ar), 128.62–127.82 (12 × C-Ar), 106.56 (d, ^1^*J*_1,F_ = 223.7 Hz, C-1β), 106.37 (d, ^1^*J*_1,F_ = 224.8 Hz, C-1α), 76.55 (C-3β), 75.46 (d, ^2^*J*_2β,F_ = 23.9 Hz, C-2β), 75.34 (C-3α), 73.99 (O*C*H_2_), 73.92 (d, ^2^*J*_2α,F_ = 29.8 Hz, C-2α), 73.59, 73.14, 72.79 (3 × O*C*H_2_), 62.54 (d, ^3^*J*_1,F_ = 3.6 Hz, C-5β), 59.78 (d,^3^*J*_1,F_ = 3.2 Hz, C-5α), 59.59 (C-4β), 55.03 (C-4α) ppm. HRMS (ESI-TOF): calcd. for C_19_H_20_FN_3_O_3_[M + Na]^+^ 380.1381; found 380.1377.

**4-Azido-2,3-di-*O*-benzyl-4-deoxy-α-l-arabinopyranosyl Trichloroaceteimidate (5a) and 4-Azido-2,3-di-*O*-benzyl-4-deoxy-β-l-arabinopyranosyl Trichloroacetimidate (5b):** Compound **3** (200 mg, 0.56 mmol) was dissolved in dry CH_2_Cl_2_ (5 mL). K_2_CO_3_ (285 mg, 2.06 mmol) was added followed by trichloroacetonitrile (282 μL, 2.81 mmol) and the suspension was stirred for 12 h at room temp. The suspension was filtered, the filtrate was concentrated, and the residue was chromatographed over deactivated silica gel (containing 1 % triethylamine). Elution of the column (hexane/EtOAc, 3:1+1 % triethylamine) afforded **5b** as the fastest moving isomer (55 mg, 19 %). [*α*]_D_^20^ = +71 (*c* = 1.2 CHCl_3_). ^1^H NMR (300 MHz, CDCl_3_): *δ* = 8.58 (br. s, 1 H, NH), 7.39–7.26 (m, 10 H, 2 × Ph), 6.41 (d, *J*_1,2_ = 2.9 Hz, 1 H, 1-H), 4.85–4.73 (m, 4 H, 2 × CH_2_Ph), 4.11 (dd, *J*_2,3_ = 9.5 Hz, 1 H, 2-H), 4.06 (dd, *J*_3,4_ = 2.8 Hz, 1 H, 3-H), 3.97 (dd, *J*_5a,5b_ = 12.9, *J*_5a,4_ = 1.8 Hz, 1 H, 5a-H), 3.96 (br. s, 1 H, 4-H), 3.77 (dd, *J*_5b,4_ = 2.5 Hz, 1 H, 5b-H) ppm. ^13^C NMR (75 MHz, CDCl_3_): *δ* = 161.09 (C=NH), 138.05, 137.74, 128.45, 128.35, 127.87, 127.67, 127.47 (C-Ar), 95.09 (C-1), 76.13 (C-2), 75.51 (C-3), 73.22, 73.16 (2 × *C*H_2_Ph), 63.05 (C-5), 59.77 (C-4) ppm.

Further elution of the column gave **5a** as a colorless amorphous solid (166 mg, 59 %). [*α*]_D_^20^ = +12 (*c* = 1.6, CHCl_3_). ^1^H NMR (300 MHz, CDCl_3_): *δ* = 8.63 (br. s, 1 H, NH), 7.38–7.28 (m, 10 H, 2 × Ph), 5.88 (d, *J*_1,2_ = 4.8 Hz, 1 H, H-1), 4.83–4.62 (m, 4 H, 2 × CH_2_Ph), 4.14 (dd, *J*_5a,5b_ = 11.7, *J*_5a,4_ = 6.0 Hz, 1 H, 5a-H), 3.93 (dd, *J*_2,3_ = 6.1 Hz, 1 H, 2-H), 3.83 (dd, *J*_3,4_ = 3.2 Hz, 1 H, 3-H), 3.81 (m, 1 H, 4-H), 3.67 (dd, *J*_5b,4_ = 3.2 Hz, 1 H, 5b-H). ^13^C NMR (75 MHz, CDCl_3_): *δ* = 161.27 (C=NH), 137.48, 137.33, 128.59–127.85 (C-Ar), 96.76 (C-1), 77.39 (C-3), 75.10 (C-2), 74.27, 72.77 (2 × *C*H_2_Ph), 62.08 (C-5), 56.76 (C-4) ppm.

**4-Azido-2,3-di-*O*-benzyl-4-deoxy-α-l-arabinopyranosyl *N*-Phenyltrifluoroacetimidate (6a) and 4-Azido-2,3-di-*O*-benzyl-4-deoxy-β-l-arabinopyranosyl *N*-Phenyltrifluoroacetimidate (6b):** A suspension of **3** (1 g, 2.81 mmol), K_2_CO_3_ (780 mg, 5.63 mmol), and *N*-phenyl-2,2,2-trifluoroacetimidoyl chloride (780 mg, 5.63 mmol; prepared according to ref.^32^) in acetone (10 mL) was stirred for 12 h at room temp. The suspension was filtered. The filtrate was concentrated and the residue was chromatographed with silica gel. Elution with hexane/EtOAc (20:1) afforded **6a** (510 mg, 34 %) as a colorless syrup. [*α*]_D_^20^ = +48 (*c* = 0.5, CHCl_3_). ^1^H NMR (400 MHz, CDCl_3_): *δ* = 7.40–7.22 (m, 12 H, Ph-H), 7.08 [t, *J* = 7.6 Hz, 1 H, *N*-Ph (H-*p*)], 6.73 [d, 2 H, *N*-Ph (2 × *o*-H)], 6.40 (br. s, 1 H, 1-H), 4.86–4.67 (m, 4 H, 2 × C*H*_2_Ph), 4.10–3.97 (m, 2 H, 2-H, 3-H), 3.97–3.69 (m, 3 H, 4-H, 5a-H, 5b-H) ppm. ^13^C NMR (100 MHz, CDCl_3_): *δ* = 143.48 (*N*-Ph C*q*), 137.87, 137.71, 128.70, 128.48, 128.42, 127.91, 127.81, 127.77, 127.52 (C-Ar), 124.25 (*N-*Ph C-*p*), 119.41 (*N-*Ph C-*o*), 76.44 (C-2), 75.31 (C-3), 73.63 and 73.24 (*C*H_2_Ph), 62.95 (C-5) and 59.62 (C-4) ppm.

Further elution gave **6b** (752 mg, 50 %) as a colorless syrup. [*α*]_D_^20^ = +89 (*c* = 1.1, CHCl_3_). ^1^H NMR (400 MHz, CDCl_3_): *δ* = 7.35–7.25 (m, 12 H, Ph-H), 7.09 [t, *J* = 7.4 Hz, 1 H, *N*-Ph (H-*p*)], 6.79 [d, 2 H, *N*-Ph (2 × *o*-H)], 5.75 (br. s, 1 H, 1-H), 4.75–4.65 (m, 4 H, 2 × C*H*_2_Ph), 4.05 (br. s, 1 H, 5a-H), 3.88 (br. s, 1 H, 2-H), 3.78 (br. s, 2 H, 3-H, 4-H), 3.54 (br. s, 1 H, 5b-H) ppm. ^13^C NMR (100 MHz, CDCl_3_): *δ* = 143.49 (*N*-Ph C*q*), 137.39, 137.32, 128.70, 128.51, 128.49, 128.07, 128.07, 128.06 and 127.91 (C-Ar), 124.28 (*N*-Ph C*p*), 119.38 (*N*-Ph C*o*), 78.04 (C-3), 75.33 (C-2), 74.51 and 72.95 (2 × O*C*H_2_), 62.50 (C-5) and 59.91 (C-4) ppm.

**2,3-Di-*O*-benzyl-4-deoxy-4-trifluoroacetamido-α-l-arabinopyranosyl Fluoride (7a) and 2,3-Di-*O*-benzyl-4-deoxy-4-trifluoroacetamido-β-l-arabinopyranosyl Fluoride (7b):** Trifluoroacetic anhydride (193 μL, 1.39 mmol) was added to a solution of **4** (250 mg, 0.69 mmol) in dry CH_2_Cl_2_ (10 mL). Triethylamine (193 μL, 1.39 mmol) and a solution of PPh_3_ (365 mg, 1.39 mmol) in dry toluene (1 mL) were added slowly. The solution was stirred for 1 h at room temp., and diluted with CH_2_Cl_2_ (50 mL). The solution was washed with saturated aqueous NaHCO_3_ solution, dried (MgSO_4_), and concentrated. The residue was purified by column chromatography on silica gel (hexane/EtOAc, 9:1). Pooling of the fractions containing the least polar compound gave 2,3-di-*O*-benzyl-4,5-dideoxy-l-*threo*-pent-4-enopyranosyl fluoride (**8**) as a colorless syrup (28 mg, 9 %). ^1^H NMR (400 MHz, CDCl_3_): *δ* = 7.38–7.26 (m, 10 H, Ph-H), 6.18 (ddd, *J*_5,4_ = 6.2, *J*_5,3_ = 1.7 Hz, 1 H, 5-H), 5.60 (d, *J*_1,F_ = 49.5, *J*_1,2_ = 1.9 Hz, 1 H, 1-H), 5.02 (dd, *J*_3,4_ = 2.5 Hz, 1 H, 4-H), 4.78 and 4.66 (2 × d, each 2 H, C*H*_2_Ph), 4.33 (ddd, 1 H, 3-H) and 3.81 (ddd, *J*_2,F_ = 24, *J*_3,2_ = 7.8 Hz, 1 H, 2-H) ppm. ^13^C NMR (HSQC): *δ* = 139.05 (C-5), 130.36–124.91 (C-Ar), 104.22 (C-1), 102.56 (C-4), 76.01 (C-2), 73.14 (*C*H_2_Ph), 72.32 (C-3), 71.7 (*C*H_2_Ph) ppm.

Further elution of the column afforded 2,3-di-*O*-benzyl-4-deoxy-d-*glycero*-pent-3-enopyranosyl fluoride (**9**) as a colorless syrup (77 mg, 26 %). ^1^H NMR (400 MHz, CDCl_3_): *δ* = 7.39–7.26 (m, 10 H, Ph-H), 5.65 (d, 1 H, *J*_1,F_ = 52.7, *J*_1,2_ = 3.3 Hz, 1-H), 4.92–4.73 (m, 5 H, 4-H, 2 × C*H*_2_Ph), 4.53 (dq, 1 H, *J*_5a,5b_ = 14.8 Hz, 5a-H), 4.22 (ddd, 1 H, *J*_5b,4_ = 3.4 Hz, 5b-H) and 4.16 (m, 1 H, *J*_2,F_ = 18.7 Hz, 2-H). ^13^C NMR (100 MHz, CDCl_3_): *δ* = 148.94 (C-3), 138.05, 136.53 and 128.58–127.39 (C-Ar), 105.13 (d, *J*_1,F_ = 226.3 Hz, C-1), 94.55 (C-4), 73.66 (O*C*H_2_), 70.73 (d, *J*_2,F_ = 23.5 Hz, C-2), 69.21 (*C*H_2_Ph) and 61.20 (d, *J*_5,F_ = 5.0 Hz, C-5).

Continued elution furnished **7a** (87 mg, 29 %) as a colorless syrup. [*α*]_D_^20^ = –105.5 (*c* = 0.3, CHCl_3_). ^1^H NMR (400 MHz, CDCl_3_): *δ* = 7.42–7.19 (m, 10 H, Ph-H), 6.49 (d, *J*_NH,4_ = 8.3 Hz, 1 H, NH), 5.48 (d, *J*_1,F_ = 49.5 Hz, 1 H, 1-H), 4.69–4.55 (m, 3 H, 1.5 × C*H*_2_Ph), 4.48 (m, 1 H, 4-H), 4.31 (d, 1 H, 0.5 × C*H*_2_Ph), 3.92 (dt, *J*_5a,5b_ = 10.6 Hz, 1 H, 5a-H), 3.76 (ddd, 1 H, 2-H), 3.71–3.66 (m, 2 H, 3-H, 5b-H) ppm. ^13^C NMR (100 MHz, CDCl_3_): *δ* = 136.72, 136.56, 128.85, 128.73, 128.22, 128.03, 127.60, 127.43 (C-Ar), 105.05 (d, *J*_1,F_ = 225.3 Hz, C-1), 73.09 (*C*H_2_Ph), 72.58 (C-3), 71.83 (*C*H_2_Ph), 70.34 (d, *J*_2,F_ = 32.3 Hz, C-2), 58.07 (d, *J*_5,F_ = 3.0 Hz, C-5) and 44.04 (C-4) ppm. HRMS (ESI-TOF): calcd. for C_21_H_21_F_4_NO_4_ [M +Na]^+^ calcd: 450.1299; found 450.1304.

Finally, elution of the column gave **7b** (55 mg, 18 %) as a syrup.[*α*]_D_^20^ = +12.2 (*c* = 0.3, CHCl_3_). ^1^H NMR (400 MHz, CDCl_3_): *δ* = 7.40–7.27 (m, 10 H, Ph-H), 6.53 (br. s, 1 H, NH), 5.48 (dd, *J*_1,F_ = 52.4, *J*_1,2_ = 2.7 Hz, 1 H, 1-H), 4.85–4.59 (m, 4 H, 2 × C*H*_2_Ph), 4.47 (m, 1 H, 4-H), 4.04 (dd, *J*_5a,4_ = 2.3, *J*_5a,5b_ = 12.9 Hz, 1 H, 5a-H), 4.03 (dd, *J*_3,2_ = 9.2 Hz, 1 H, 3-H), 3.92 (dd, *J*_5b,4_ = 2.4 Hz, 1 H, 5b-H) and 3.76 (ddd, *J*_2,F_ = 23.5 Hz, 1 H, 2-H) ppm. ^13^C NMR (100 MHz, CDCl_3_): *δ* = 137.52, 137.06, 128.59, 128.55, 128.18, 128.11, 128.08, 127.93 (C-Ar), 105.74 (d*, J*_1,F_ = 228.3 Hz, C-1), 74.43 (C-3), 74.42 (*J*_2,F_ = 23.6 Hz, C-2), 73.88 and 72.28 (2 × *C*H_2_Ph), 61.58 (d, *J*_5,F_ = 5.0 Hz, C-5), and 48.16 (C-4) ppm. HRMS (ESI-TOF): calcd. for C_21_H_21_F_4_NO_4_ [M + Na]^+^ 450.1299; found 450.1292.

**Methyl (Allyl 3,4,5,7-tetra-*O*-acetyl-8-*O*-*tert*-butyldimethylsilyl-d-*glycero*-α-d-*talo*-oct-2-ulopyranoside)onate (14):** A solution of methyl (allyl 3,4,5,7,8-penta-*O*-acetyl-d-*glycero*-α-d-*talo*-oct-2-ulopyranoside)onate (250 mg, 0.48 mmol) in dry MeOH (5 mL) was stirred with methanolic NaOMe (0.25 mL, 0.1 M) for 3 h at room temp. The solution was deionized with Dowex 50 (H^+^) cation-exchange resin, filtered, and the filtrate was concentrated. The residue was dried and dissolved in dry acetonitrile (5 mL). DABCO(65 mg, 0.58 mmol) and (*tert*-butyl)chlorodimethylsilane (87 mg, 0.58 mmol) were added. The mixture was stirred at room temp. for 15 h. The suspension was concentrated and the residue was purified by flash chromatography (EtOAc). The product was taken up in dry pyridine (5 mL) and cooled to –10 °C. A catalytic amount of DMAP was added followed by a mixture of pyridine/Ac_2_O (1:1, 4 mL). The solution was stirred for 12 h at room temp., cooled to 0 °C, and dry MeOH (3 mL) was added. Stirring was continued at 0 °C for a further 15 min. The solution was coevaporated several times with toluene, and the residue was taken up in chloroform (50 mL). The organic layer was washed with saturated aqueous NaHCO_3_ solution and dried (MgSO_4_). Concentration left a syrup, which was purified on a column of silica gel (toluene/EtOAc, 5:1 + 1 % triethylamine) to afford **14** (179 mg, 62 %) as a colorless syrup. [*α*]_D_^20^ = +26 (*c* = 1.8, CHCl_3_). ^1^H NMR (300 MHz, CDCl_3_): *δ* = 5.89–5.75 (m, 1 H, –C*H*=), 5.52 (dd, *J*_3,4_ = 3.7, *J*_3,5_ = 0.9 Hz, 1 H, 3-H), 5.43 (t, *J*_4,5_ = 3.7 Hz, 1 H, 4-H), 5.35 (ddd, *J*_5,6_ = 1.7 Hz, 1 H, 5-H), 5.27 [dq, 1 H, =CH_2_(*trans*)], 5.21–5.15 (m, 2 H, =CH_2*cis*_, 7-H), 4.32 (dd, *J*_6,7_ = 9.9 Hz, 1 H, 6-H), 4.23 (dq, 1 H, OC*H*_2_), 4.06 (dd, *J*_8a,8b_ = 11.9, *J*_8a,7_ = 2.2 Hz, 1 H, 8a-H), 3.89–3.80 (m, 2 H, OC*H*_2_, 8b-H), 3.75 (s, 3 H, CO_2_CH_3_), 2.05 (s 3 H), 2.04 (s, 3 H), 1.99 (s, 3 H) and 1.96 (s, 3 H, 4 × COC*H*_3_), 0.87 [s, 9 H, C(C*H*_3_)_3_], 0.04 (s, 3 H) and 0.01 [s, 3 H, Si(C*H*_3_)_2_] ppm. ^13^C NMR (75 MHz, CDCl_3_): *δ* = 170.34, 169.79, 169.36, 168.95 (4 × *C*OCH_3_), 166.01 (C-1), 132.59 (–*C*H=), 117.54 (=*C*H_2_), 99.08 (C-2), 70.15 (C-7), 67.66 (C-3), 67.02 (C-6), 66.16 (C-4), 65.08 (O*C*H_2_), 64.20 (C-5), 61.12 (C-8), 52.61 (CO_2_*C*H_3_), 25.84 [*C*(CH_3_)_3_], 20.80, 20.65, 20.52, 20.49 (4 × CO*C*H_3_) and 18.43 [C(*C*H_3_)_3_] ppm. HRMS (ESI-TOF): calcd. for C_26_H_42_O_13_Si [M + Na]^+^ 613.2287; found 613.2280.

**Desilylation of 10:** A solution of **10** (736 mg, 1.38 mmol) was treated with a solution of 2 % HF in MeCN (0.6 mL) in dry acetonitrile (5 mL) at room temp. for 2 h. Solid NaHCO_3_ was added to the solution and stirring was continued for 20 min. The suspension was filtered, and the filtrate was concentrated, coevaporated with toluene three times, and thoroughly dried to give **11** (578 mg, 99 %) as a syrup. The material was immediately used for the subsequent glycosylation step.

**Desilylation of 14:** A solution of **14** (550 mg, 0.93 mmol) was treated with a solution of 2 % HF in MeCN (0.6 mL) in dry acetonitrile (10 mL) at room temp. for 2 h. Solid NaHCO_3_ was added to the solution and stirring was continued for 20 min. The suspension was filtered, and the filtrate was concentrated, coevaporated with toluene three times, and thoroughly dried to give **15** (443 mg, 99 %) as a syrup. The material was immediately used for the subsequent glycosylation step.

**Methyl 4-Azido-2,3-di-*O*-benzyl-4-deoxy-β-l-arabinopyranosyl-(1→8)-(allyl 4,5,7-tri-*O*-acetyl-3-deoxy-α-d-*manno*-oct-2-ulopyranoside)onate (16) and Methyl 4-Azido-2,3-di-*O*-benzyl-4-deoxy-α-l-arabinopyranosyl-(1→8)-(allyl 4,5,7-tri-*O*-acetyl-3-deoxy-α-d-*manno*-oct-2-ulopyranoside)onate (18):** A solution of **11** (578 mg, obtained by desilylation of **10**) in dry CH_2_Cl_2_ (5 mL) was added to a suspension of **6** (1.45 g, 2.76 mmol) in dry CH_2_Cl_2_ (20 mL) with 4 Å molecular sieves. The suspension was stirred for 2 h at –15 °C under Ar. A solution of TMSOTf (12.5 μL, 69 μmol) in dry CH_2_Cl_2_ (2 mL) was slowly added and stirring was continued for 1 h with gradual warming to room temp. The reaction was quenched by adding triethylamine (50 μL). The suspension was diluted with CH_2_Cl_2_ (20 mL), filtered through a short plug of Celite®, and washed with CH_2_Cl_2_. The filtrate was washed with saturated aqueous NaHCO_3_, dried (MgSO_4_), and concentrated. The crude material (2 g) was directly applied to column chromatography (toluene/EtOAc, 3:1), which gave a mixture of **16/19** (550 mg, 52 %) and **18** (230 mg, 22 %) as a colorless syrup. [*α*]_D_^20^ = +45 (*c* = 0.5, CHCl_3_). ^1^H NMR (400 MHz, CDCl_3_): *δ* = 7.36–7.27 (m, 10 H, Ph-H), 5.75 (m, 1 H, –C*H*=), 5.33 (dd, *J*_4,5_ = 3.0, *J*_6,5_ = 1.4 Hz, 1 H, 5-H), 5.29 (ddd, *J*_4,3e_ = 5.0, *J*_4,3a_ = 12.0 Hz, 1 H, 4-H), 5.24–5.18 [m, 2 H, 7-H, =CH_2_(*trans*)], 5.12 [dq, 1 H, =CH_2_(*cis*)], 4.74–4.63 (m, 4 H, 2 × C*H*_2_Ph), 4.32 (d, *J*_1′,2′_ = 6.2 Hz, 1 H, 1′-H), 4.10 (dd, *J*_8a,7_ = 2.4, *J*_8a,8b_ = 11.2 Hz, 1 H, 8a-H), 4.06 (dd, *J*_6,7_ = 9.6 Hz, 1 H, 6-H), 4.00 (m, 1 H, OC*H*_2_), 3.94 (dd, *J*_5′a,5′b_ = 12.1, *J*_4′,5′a_ = 4.1 Hz, 1 H, 5′a-H), 3.87 (m, 1 H, OC*H*_2_), 3.78 (s, 3 H, CO_2_C*H*_3_), 3.77–3.72 (m, 2 H, 8b-H, 4′-H), 3.68–3.62 (m, 2 H, 2′-H, 3′-H), 3.42 (dd, *J*_4′,5′b_ = 2.2 Hz, 1 H, 5′b-H), 2.18 (ddd, *J*_3*e*,3*a*_ = 12.7 Hz, 1 H, 3*e*-H), 2.09–2.04 (m, 4 H, 3*a*-H, COC*H*_3_), 1.97 (s, 3 H) and 1.90 (s, 3 H, 2 × COC*H*_3_) ppm. ^13^C NMR (100 MHz, CDCl_3_): *δ* = 170.46–169.79 (3 × O*C*CH_3_), 167.77 (C-1), 138.07, 137.61 (2 × C-Ar), 133.28 (–*C*H=), 128.44, 128.37, 127.90, 127.80, 127.78 and 127.71 (6 × C-Ar), 117.26 (=*C*H_2_), 102.85 (C-1′), 98.53 (C-2), 78.99 (C-3′), 77.71 (C-2′), 74.63 and 72.71 (2 × O*C*H_2_Ph), 68.83 (C-7), 68.73 (C-6), 67.34 (C-8), 66.41 (C-4), 64.77 (O*C*H_2_), 64.49 (C-5), 62.38 (C-5′), 57.94 (C-4′), 52.62 (OCH_3_), 31.93 (C-3), 20.84–20.67 (3 × OC*C*H_3_) ppm. HRMS (ESI-TOF): calcd. for C_37_H_45_N_3_O_14_ [M + HCOO^–^]^–^ 800.2884; found 800.2890.

**Methyl 4-Azido-2,3-di-*O*-benzyl-4-deoxy-β-l-arabinopyranosyl-(1→8)-(allyl 3-deoxy-α-d-*manno*-oct-2-ulopyranoside)onate (17) and Methyl 4-Azido-2,3-di-*O*-benzyl-4-deoxy-β-l-arabinopyranosyl-(1→7)-(allyl 3-deoxy-α-d-*manno*-oct-2-ulopyranoside)onate (20):** A solution of **16**/**19** (550 mg, 0.727 mmol) in dry MeOH (5 mL) was stirred with NaOMe (0.5 mL, 0.1 M) for 4 h at room temp. The solution was neutralized by addition of Dowex 50 (H^+^) cation-exchange resin and filtered. The filtrate was concentrated. Purification of the residue by column chromatography (toluene/EtOAc, 1:2) first afforded **20** (138 mg, 30 %). [*α*]_D_^20^ = +107 (*c* = 0.9, CHCl_3_). ^1^H NMR (400 MHz, CDCl_3_): *δ* = 7.43–7.29 (m, 10 H, Ph-H), 5.85 (m, 1 H, –C*H*=), 5.25 [dq, 1 H, =CH_2_(*trans*)], 5.14 [dq, 1 H, =CH_2_(*cis*)], 5.00 (d, *J*_1′,2′_ = 3.6 Hz, 1 H, 1′-H), 4.98–4.65 (m, 4 H, 2 × C*H*_2_Ph), 4.03 (dd, *J*_3′,2′_ = 9.8, *J*_3′,4′_ = 3.5 Hz, 1 H, 3′-H), 4.00–3.62 (m, 14 H, 2′-H, 4′-H, 4-H, 5-H, 5′a-H, 5′b-H, 7-H, 8a-H, 8b-H, OCH_2_CO_2_CH_3_), 3.61 (dd, *J*_6,5_ = 1.3, *J*_6,7_ = 8.5 Hz, 1 H, 6-H), 2.14 (dd, *J*_3e,3a_ = 12.8, *J*_3e,4_ = 4.9 Hz, 1 H, 3*e*-H) and 1.72 (dd, *J*_3a,4_ = 11.8 Hz, 1 H, 3*a*-H) ppm. ^13^C NMR (100 MHz, CDCl_3_): *δ* = 168.41 (C-1), 137.26 and 136.38 (C-Ar), 133.55 (–*C*H=), 128.71, 128.66, 128.50, 128.17, 128.01 and 127.80 (C-Ar), 116.90 (=*C*H_2_), 100.80 (C-1′), 98.89 (C-2), 81.87 (C-7), 77.18 (C-3′), 75.49 (C-2′), 75.22 and 72.32 (2 × *C*H_2_Ph), 71.07 (C-6), 65.30 (C-4), 64.72 (C-5), 64.49 (O*C*H_2_), 63.74 (C-8), 61.53 (C-5′), 59.35 (C-4′), 52.51 (CO_2_*C*H_3_) and 35.26 (C-3) ppm.

Further elution of the column with EtOAc/EtOH (2:1) gave **17** (316 mg, 69 %) as a colorless syrup. [*α*]_D_^20^ = +78.6 (*c* = 0.36, CHCl_3_). ^1^H NMR (400 MHz, CDCl_3_): *δ* = 7.34–7.22 (m, 10 H, Ph-H), 5.73 (m, 1 H, –C*H*=), 5.16 [dq, 1 H, =CH_2_(*trans*)], 5.03 [dq, 1 H, =CH_2_(*cis*)], 4.79 (d, *J*_1′,2′_ = 3.6 Hz, 1 H, 1′-H), 4.82–4.58 (m, 4 H, C*H*_2_Ph), 3.99–3.78 (m, 9 H, 2′-H, 3′-H, 4′-H, 4-H, 5-H, 7-H, 8a-H, OC*H*_2_), 3.71 (dd, *J*_5′a,4′_ = 1.7, *J*_5′a,5′b_ = 12.5 Hz, 1 H, 5′a-H), 3.69 (s, 3 H, CO_2_C*H*_3_), 3.69 (dd, *J*_8a,7_ = 3.5, *J*_8a,8b_ = 11.5 Hz, 1 H, 8b-H), 3.53 (dd, *J*_5′b,4′_ = 2.2 Hz, 1 H, 5′b-H), 3.48 (dd, *J*_6,5_ = 0.9, *J*_6,7_ = 7.8 Hz, 1 H, 6-H), 2.11 (dd, *J*_3e,3a_ = 12.8, *J*_3e,4_ = 5.3 Hz, 1 H, 3*e*-H), 1.80 (dd, *J*_3a,4_ = 11.3 Hz, 1 H, 3*a*-H) ppm. ^13^C NMR (100 MHz, CDCl_3_): *δ* = 168.53 (C-1), 137.59 and 137.52 (C-Ar), 133.67 (–*C*H=), 128.53, 128.28, 128.17, 128.00 and 127.73 (C-Ar), 116.77 (=*C*H_2_), 101.06 (C-1′), 98.82 (C-2), 77.15 (C-3′), 75.97 (C-2′), 74.46 and 72.51 (2 × *C*H_2_Ph), 71.74 (C-6), 70.64 (C-8), 69.04 (C-7), 66.57 (C-5), 65.63 (C-4), 64.27 (O*C*H_2_), 60.86 (C-5′), 59.64 (C-4′), 52.57 (CO_2_*C*H_3_) and 34.98 (C-3) ppm. HRMS (ESI-TOF): calcd. for C_31_H_39_N_3_O_11_ [M + HCOO^–^]^–^ 674.2567; found 674.2570.

**Acetylation of 17:** A solution of **17** (230 mg, 0.35 mmol) in dry pyridine (3 mL) was stirred with acetic anhydride (2 mL) in pyridine (1 mL) with a catalytic amount of DMAP for 24 h at room temp. The solution was cooled to 0 °C, methanol (3 mL) was added, and stirring was continued for 30 min. The solution was coevaporated with toluene. The residue was directly applied to column chromatography (toluene/EtOAc, 5:1) to give **16** (241 mg, 80 %) as a colorless syrup. [*α*]_D_^20^ = +83 (*c* = 0.52, CHCl_3_). ^1^H NMR (400 MHz, CDCl_3_): *δ* = 7.47–7.27 (m, 10 H, Ph-H), 5.80 (m, 1 H,–C*H*=), 5.36–5.34 (m, 1 H, 5-H), 5.32 (ddd, 1 H, 4-H), 5.25 [dq, 1 H, =CH_2_(*trans*)], 5.20 (m, 1 H, 7-H), 5.09 [dq, 1 H, =CH_2_(*cis*)], 4.89 (d, *J*_1′,2′_ = 3.3 Hz, 1 H, 1′-H), 4.77–4.64 (m, 4 H, 2 × C*H*_2_Ph), 4.21 (dd, *J*_6,5_ = 1.3 Hz, 1 H, 6-H), 4.15–4.09 (m, 1 H, OC*H*_2_), 3.91–3.84 (m, 4 H, 8a-H, 8b-H, 3′-H, OC*H*_2_), 3.82 (dd, *J*_2′,1′_ = 3.3, *J*_2′,3′_ = 12.5 Hz, 1 H, 2′- H), 3.82 (m, 1 H, 4′-H), 3.79 (s, 3 H, CO_2_C*H*_3_), 3.72 (dd, *J*_5′a,5′b_ = 12.3, *J*_4′,5′a_ = 1.9 Hz, 1 H, 5′a-H), 3.57 (dd, *J*_4′,5′b_ = 2.8 Hz, 1 H, 5′b-H), 2.21 (ddd, *J*_3*e*,3*a*_ = 12.5 Hz, 1 H, 3*e*-H), 2.05 (t, 1 H, 3*a*-H), 2.08 (s, 3 H), 1.99 (s, 3 H), and 1.92 (s, 3 H, 3 × COC*H*_3_) ppm. ^13^C NMR (100 MHz, CDCl_3_): *δ* = 170.50, 169.81 and 169.61 (3 × O*C*CH_3_), 167.81 (C-1), 138.21, 137.94 (2 × C-Ar), 133.52 (–*C*H=), 128.5–127.5 (10 × C-Ar), 117.60 (=*C*H_2_), 98.51 (C-2), 98.45 (C-1′), 76.95 (C-3′), 75.96 (C-2′), 73.61 and 73.13 (2 × O*C*H_2_Ph), 69.38 (C-5), 68.39 (C-6), 66.47 (C-4), 65.73 (C-8), 65.01 (O*C*H_2_), 64.50 (C-7), 60.77 (C-5′), 59.80 (C-4′), 52.63 (OCH_3_), 31.96 (C-3), 20.78, 20.78, 20.72 (3 × OC*C*H_3_) ppm. HRMS (ESI-TOF): calcd. for C_37_H_45_N_3_O_14_ [M + HCOO^–^]^–^ 800.2884; found 800.2890.

**Methyl 2,3-Di-*O*-acetyl-4-azido-4-deoxy-β-l-arabinopyranosyl-(1→8)-(allyl 4,5,7-tri-*O*-acetyl-3-deoxy-α-d-*manno*-oct-2-ulopyranoside)onate (21):** A solution of **16** (56 mg, 0.074 mmol) in dry chloroform (5 mL) was stripped with argon. A solution of TiCl_4_ (17.2 μL, 0.156 mmol) in chloroform (1 mL) was added slowly at 0 °C. After 1 h, additional TiCl_4_ (17.2 μL) was added until the TLC showed the complete consumption of starting material and the appearance of a lower migrating compound (*R*_f_ 0.18, EtOAc/toluene, 1:1). Ethyl ether (10 mL) and saturated aqueous NaHCO_3_ solution (5 mL) were added with caution and the solution was stirred for 30 min. The organic layer was dried (MgSO_4_), and concentrated. The residue was purified by flash chromatography over silica gel (toluene/EtOAc, 1:1) to give the crude debenzylated product. A solution of this residue in pyridine (2 mL) and a catalytic amount of DMAP was cooled to 0 °C. A solution of acetic anhydride in pyridine (2 mL, 2:1) was added slowly and the reaction was stirred for 24 h at room temp. Dry MeOH (2 mL) was added at 0 °C and stirring was continued for 30 min. The solution was coevaporated with toluene five times and concentrated. The residue was purified by column chromatography (toluene/EtOAc, 4:1) to furnish **21** (33 mg, 68 %) as a colorless syrup. [*α*]_D_^20^ = +72 (*c* = 0.3, CHCl_3_). ^1^H NMR (600 MHz, CDCl_3_): *δ* = 5.93 (m, 1 H, –C*H*=), 5.36–5.30 [m, 3 H, 4-H, 5-H, =C*H*_2_(*trans*)], 5.23 (dd, *J*_2′,1′_ = 3.7, *J*_2′,3′_ = 10.5 Hz, 1 H, 3′-H), 5.20 [dq, 1 H, =C*H*_2_(*cis*)], 5.15 (ddd, *J*_7,8a_ = 3.3, *J*_7,8b_ = 2.2, *J*_7,6_ = 9.8 Hz, 1 H, 7-H), 5.15 (dd, *J*_2′,1′_ = 3.7, *J*_2′,3′_ = 10.5 Hz, 1 H, 2′-H), 5.12 (d, *J*_1′,2′_ = 3.7 Hz, 1 H, 1′-H), 4.19 (dd, *J*_6,5_ = 1.3 Hz, 1 H, 6-H), 4.16–4.12 (ddt, 1 H, OC*H*_2_), 4.09 (dt, *J*_4′,5′a_ = 1.8 Hz, 1 H, 4′-H), 4.00–3.96 (ddt, 1 H, OC*H*_2_), 3.95 (dd, *J*_8a,8b_ = 12.9 Hz, 1 H, 8a-H), 3.90 (dd, *J*_5′a,5′b_ = 12.6 Hz, 1 H, 5′a-H), 3.81 (s, 3 H, CO_2_C*H*_3_), 3.80 (dd, 1 H, 8b-H), 3.66 (dd, 1 H, 5′b-H), 2.22 (ddd, *J*_3*e*,3*a*_ = 12.5, *J*_3e,4_ = 4.9 Hz, 1 H, 3*e*-H), 2.09 (t, *J*_3a,4_ = 11.8 Hz, 1 H, 3*a*-H), 2.13 (s, 3 H), 2.12 (s, 3 H), 2.07 (s, 3 H), 2.00 (s, 3 H) and 1.97 (s, 3 H, 5 × C*H*_3_CO) ppm. ^13^C NMR (150 MHz, CDCl_3_): *δ* = 170.47, 170.13, 170.09, 169.94, and 169.56 (5 × O*C*CH_3_), 167.71 (C-1), 133.25 (–*C*H=), 117.22 (=*C*H_2_), 98.58 (C-2), 97.29 (C-1′), 69.13 (C-2′), 69.09 (C-3′), 68.17 (C-6), 68.10 (C-7), 66.59 (C-4), 65.82 (C-8), 64.79 (O*C*H_2_), 64.38 (C-5), 60.05 (C-5′), 59.61 (C-4′), 52.7 (CO_2_CH_3_), 31.95 (C-3), 20.73, 20.71, 20.66, 20.55, 20.53 (5 × OC*C*H_3_) ppm. HRMS (ESI-TOF): calcd. for C_27_H_37_N_3_O_16_ [M + Na]^+^ 682.2066; found 682.2075.

**Sodium 4-Azido-4-deoxy-β-l-arabinopyranosyl-(1→8)-(allyl 3-deoxy-α-d-*manno*-oct-2-ulopyranoside)onate (22):** A solution of **21** (33 mg, 0.045 mmol) in dry methanol (2 mL) was stirred with methanolic NaOMe (0.1 mL, 0.1 M) for 4 h at room temp. The solution was neutralized with Dowex 50 (H^+^) cation-exchange resin. The suspension was filtered, and the filtrate was concentrated. A solution of the residue in water (2 mL) was then adjusted to pH 12 with aqueous NaOH (ca. 2 mL, 0.2 M) and stirred at room temp. for 3 h. The solution was neutralized with Dowex 50 (H+) cation-exchange resin. The resin was removed by filtration and the filtrate was lyophilized to give **22** (20 mg, 98 %) as a colorless syrup. [*α*]_D_^20^ = +112 (*c* = 0.47, MeOH). ^1^H NMR (400 MHz, CD_3_OD): *δ* = 5.96 (m, 1 H, –C*H*=), 5.31–5.24 [dq, 1 H, =C*H*_2_(*trans*)], 5.14–5.08 [dq, 1 H, =C*H*_2_(*cis*)], 4.83 (d, *J*_1′,2′_ = 3.6 Hz, 1 H, 1′-H), 4.06 (ddd, *J*_7,8a_ = 5.2, *J*_7,8b_ = 3.3, *J*_7,6_ = 7.9 Hz, 1 H, 7-H), 4.02 (dd, *J*_3′,4′_ = 3.9, *J*_2′,3′_ = 9.7 Hz, 1 H, 3′-H), 3.99–3.94 (m, 4 H, 4-H, 5-H, OC*H*_2_), 3.92 (dd, *J*_5′a,5′b_ = 12.6 Hz, 1 H, 5′a-H), 3.89 (dd, *J*_8a,8b_ = 10.7 Hz, 1 H, 8a-H), 3.83 (dt, *J*_4′,5′a_ = 1.9 Hz, 1 H, 4′-H), 3.73 (dd, *J*_2′,1′_ = 3.6, *J*_2′,3′_ = 9.7 Hz, 1 H, 2′-H), 3.69 (dd, 1 H, 8b-H), 3.60 (dd, *J*_6,5_ = 0.9 Hz, 1 H, 6-H), 3.59 (dd, 1 H, 5′b-H), 2.09 (ddd, *J*_3*e*,3*a*_ = 12.5, *J*_3e,4_ = 5.0 Hz, 1 H, 3*e*-H), 1.84 (dd, *J*_3a,4_ = 11.5 Hz, 1 H, 3*a*-H) ppm. ^13^C NMR (100 MHz, CD_3_OD): *δ* = 175.83 (C-1), 136.37 (–*C*H=), 116.25 (=*C*H_2_), 101.79 (C-2), 101.66 (C-1′), 73.55 (C-6), 71.59 (C-8), 70.73 (d.i. C-2′, C-3′), 69.69 (C-7), 68.67 (C-5), 67.85 (C-4), 65.15 (O*C*H_2_), 64.00 (C-4′), 61.87 (C-5′) and 36.24 (C-3) ppm. HRMS (ESI-TOF): calcd. for C_27_H_37_N_3_O_16_ [M + Na]^+^ 458.1381; found 458.1387.

**Sodium 4-Amino-4-deoxy-β-l-arabinopyranosyl-(1→8)-(allyl 3-deoxy-α-d-*manno*-oct-2-ulopyranoside)onate (23):** Compound **22** (20 mg, 0.045 mmol) was dissolved in a solution of H_2_O/diisopropylamine (2 mL, 3:1) and stripped with argon in a light-protected flask. Dithiothreitol (29.8 mg, 0.183 mmol) was added and the solution was stirred for 2 h at room temp. under argon. Another portion of dithiothreitol (29.8 mg) was added to complete the conversion. The solution was diluted with H_2_O (2 mL) and washed with chloroform (2 mL) five times. The organic phases were reextracted with water. The combined aqueous phases were stripped with argon until the solution became clear and was lyophilized. The residue was dissolved in H_2_O (1 mL) and rinsed through a column of Dowex 50 (H^+^) cation-exchange resin. The column was washed with water (20 mL) and the product was eluted with ammonia (20 mL, 1 M). The appropriate fractions were pooled and lyophilized to give **23** (12 mg, 60 %) as a white amorphous solid.[*α*]_D_^20^ = +83 (*c* = 0.29, H_2_O). ^1^H NMR (400 MHz, D_2_O, pD 7.5): *δ* = 5.93 (m, 1 H, –C*H*=), 5.35–5.28 [dq, 1 H, =C*H*_2_(*trans*)], 5.23–5.18 [dq, 1 H, =C*H*_2_(*cis*)], 4.99 (d, *J*_1′,2′_ = 3.7 Hz, 1 H, 1′-H), 4.11–4.03 (m, 3 H, 3′-H, 4-H, 7-H), 4.01 (br. s, 1 H, 5-H), 3.99 (dd, *J*_5′a,5′b_ = 11.5, *J*_5′a,4′_ = 2.0 Hz, 1 H, 5′a-H), 3.94–3.78 (m, 4 H, 8a-H, 8b-H, OC*H*_2_), 3.74 (dd, *J*_2′,1′_ = 3.7, *J*_2′,3′_ = 9.9 Hz, 1 H, 2′-H), 3.68 (dd, 1 H, 5′b-H), 3.61 (dd, *J*_6,5_ = 0.9, *J*_6,7_ = 9.3 Hz, 1 H, 6-H), 3.49 (br. s, 1 H, 4′-H), 2.03 (dd, *J*_3*e*,3*a*_ = 13.0, *J*_3e,4_ = 4.8 Hz, 1 H, 3*e*-H), 1.77 (t, *J*_3a,4_ = 13.0 Hz,1 H, 3*a*-H) ppm. ^13^C NMR (100 MHz, D_2_O): *δ* = 176.00 (C-1), 134.60 (–*C*H=), 118.50 (=*C*H_2_), 100.80 (C-2), 99.92 (C-1′), 72.33 (C-6), 70.84 (C-8), 68.90, 68.53 (C-2′, C-3′), 67.23 (C-7), 67.03 (C-4), 66.79 (C-5), 65.19 (O*C*H_2_), 60.07 (C-5′), 52.30 (C-4′) and 34.92 (C-3) ppm. HRMS (ESI-TOF): calcd. for C_16_H_27_NO_11_ [M + H]^+^ 410.1657; found 410.1659.

**Ammonium 4-Amino-4-deoxy-β-l-arabinopyranosyl-(1→8)-[3-(3-mercaptopropylthio)propyl 3-deoxy-α-d-*manno*-oct-2-ulopyranoside]onate (24):** Propane-1,3-dithiol (0.1 mL) was added to a solution of **23** (2.6 mg, 6.3 μmol) in MeOH (2 mL) and stripped with argon in a quartz flask. The reaction mixture was stirred under UV radiation (254 nm) for 1 h at room temp. to give full conversion. The solution was diluted with H_2_O (2 mL) and washed with chloroform (2 mL) five times. The organic phases were reextracted with water, and the combined aqueous phases were stripped with argon until the solution became clear. The solution was lyophilized, and the residue was purified on BioGel P-2 (5 % aq. EtOH) to give **24** (2.1 mg, 63 %) as a white solid. [*α*]_D_^20^ = +76.5 (*c* = 0.2, H_2_O). ^1^H NMR (400 MHz, D_2_O): *δ* = 5.04 (d, *J*_1′,2′_ = 3.7 Hz, 1 H, 1′-H), 4.19–4.05 (m, 5 H, 3′-H, 4-H, 5-H, 5′a-H, 7-H), 3.93 (dd, *J*_8a,8b_ = 10.6, *J*_8a,7_ = 2.7 Hz, 1 H, 8a-H), 3.86 (dd, *J*_8b,7_ = 7.5 Hz, 1 H, 8b-H), 3.80 (dd, *J*_2′,3′_ = 9.8 Hz, 1 H, 2′- H), 3.72 (dd, *J*_5′b,5′a_ = 12.9, *J*_5′b,4′b_ = 2.2 Hz, 1 H, 5′b-H), 3.61 (dd, *J*_6,7_ = 9.2 Hz, 1 H, 6-H), 3.53–3.49 (m, 2 H, 4′-H, OC*H*_2_), 3.38–3.36 (m, 1 H, OC*H*_2_), 2.73–2.64 (m, 6 H, 3 × CH_2_), 2.06 (dd, *J*_3e,3a_ = 13.1, *J*_3e,4_ = 5.0 Hz, 1 H, 3*e*-H), 1.95–1.87 (m, 4 H, 2 × CH_2_), 1.80 (t, *J*_4,3a_ = 13.1 Hz, 1 H, 3*a*-H) ppm. ^13^C NMR (100 MHz, D_2_O): *δ* = 176.28 (C-1), 101.05 (C-2), 99.83 (C-1′), 72.77 (C-6), 71.29 (C-8), 69.12 (C-2′), 68.73 (C-3′), 67.87 (C-7), 67.23 (C-4), 67.70 (C-5), 62.56 (O*C*H_2_), 60.65(C-5′), 52.38 (C-4′), 35.22 (C-3), 33.58 (OCH_2_*C*H_2_CH_2_S),30.50 (OCH_2_CH_2_*C*H_2_S), 29.57 (S*C*H_2_CH_2_CH_2_SH), 28.92 (SCH_2_*C*H_2_CH_2_SH), and 23.62 (SCH_2_CH_2_*C*H_2_SH) ppm. HRMS (ESI-TOF): calcd. for C_19_H_35_NO_11_S_2_ [M – H]^–^ 516.1579; found 516.1576.

**Methyl 4-Azido-2,3-di-*O*-benzyl-4-deoxy-β-l-arabinopyranosyl-(1→8)-(allyl 3,4,5,7-tetra-*O*-acetyl-d-*glycero*-α-d-*talo*-oct-2-ulopyranoside)onate (25) and Methyl 4-Azido-2,3-di-*O*-benzyl-4-deoxy-α-l-arabinopyranosyl-(1→8)-(allyl 3,4,5,7-tetra-*O*-acetyl-d-*glycero*-α-d-*talo*-oct-2-ulopyranoside)onate (27):** A solution of **15** (443 mg, 0.93 mmol) was taken up in dry CH_2_Cl_2_ (5 mL) and added to a suspension of **6** (970 mg, 1.86 mmol) in dry CH_2_Cl_2_ (5 mL) containing molecular sieves 4 Å. The suspension was stirred under argon at –15 °C for 2 h. A solution of TMSOTf (8.4 μL, 0.04 mmol) dissolved in dry CH_2_Cl_2_ (5 mL) was slowly added. After 1 h, triethylamine (50 μL) was added slowly. The suspension was filtered through a short plug of Celite, and the Celite bed was washed with CH_2_Cl_2_. The filtrate was washed with saturated aqueous NaHCO_3_ solution, dried (MgSO_4_), filtered, and concentrated. The crude mixture was directly applied to column chromatography (toluene/EtOAc, 3:1) to give a mixture of **25/28** (430 mg, 57 %) and **27** (284 mg, 37 %) as a colorless syrup. [*α*]_D_^20^ = +24 (*c* = 0.8, CHCl_3_). ^1^H NMR (600 MHz, CDCl_3_): *δ* = 7.38–7.27 (m, 10 H, Ph-H), 5.69 (m, 1 H, –C*H*=), 5.49 (dd, *J*_3,4_ = 3.2, *J*_3,5_ = 1.3 Hz, 1 H, 3-H), 5.32–5.27 (m, 3 H, 4-H, 5-H, 7-H), 5.19 [dq, 1 H, =C*H*_2_(*trans*)], 5.12 [dq, 1 H, =C*H*_2_(*cis*)], 4.73–4.63 (m, 4 H, 2 × C*H*_2_Ph), 4.38 (d, *J*_1′,2′_ = 5.5 Hz, 1 H, 1′-H), 4.15 (dd, *J*_6,5_ = 1.5, *J*_6,7_ = 9.7 Hz, 1 H, 6-H), 4.10 (dd, *J*_8a,7_ = 2.3, *J*_8a,8b_ = 11.4 Hz, 1 H, 8a-H), 4.06 (m, 1 H, OC*H*_2_), 3.96 (dd, *J*_5′a,4′_ = 4.5, *J*_5′a,5′b_ = 12.4 Hz, 1 H, 5′a-H), 3.88 (dd, *J*_8b,7_ = 3.9 Hz, 1 H, 8b-H), 3.79–3.72 (m, 5 H, 4′-H, CO_2_C*H*_3_, OC*H*_2_), 3.68 (dd, *J*_3′,2′_ = 7.6, *J*_3′,4′_ = 3.4 Hz, 1 H, 3′-H), 3.63 (d, 1 H, 2′-H), 3.43 (dd, *J*_4′,5′b_ = 2.4 Hz, 1 H, 5′b-H), 2.05 (s, 3 H), 2.03 (s, 3 H), 1.93 (s, 3 H) and 1.92 (s, 3 H, 4 × C*H*_3_CO) ppm. ^13^C NMR (100 MHz, CHCl_3_): *δ* = 170.29–168.97 (4 × CH_3_*C*O), 165.86 (C-1), 137.97 and 137.61 (C-Ar), 132.47 (–*C*H=), 128.48, 128.45, 127.95, 127.84 and 127.67 (C-Ar), 117.80 (=*C*H_2_), 102.56 (C-1′), 99.14 (C-2), 78.89 (C-3′), 77.50 (C-2′), 74.60 and 72.66 (*C*H_2_Ph), 68.71 (C-7), 68.15 (C-6), 67.54 (C-3), 66.91 (C-8), 66.00 (C-4), 65.09 (O*C*H_2_), 63.97 (C-5), 62.10 (C-5′), 57.72 (C-4′), 52.66 (CO_2_CH_3_), 20.74, 20.70, 20.55, and 20.51 (4 × OC*C*H_3_) ppm. HRMS (ESI-TOF): calcd. for [M + HCOO^–^]^–^ 858.2938; found 858.2946.

**Methyl 4-Azido-2,3-di-*O*-benzyl-4-deoxy-β-l-arabinopyranosyl-(1→8)-(allyl d-*glycero*-α-d-*talo*-oct-2-ulopyranoside)onate (26) and Methyl 4-Azido-2,3-di-*O*-benzyl-4-deoxy-β-l-arabinopyranosyl-(1→7)-(allyl d-*glycero*-α-d-*talo*-oct-2-ulopyranoside)onate (29):** A solution of **25/28** (430 mg, 0.528 mmol) in dry MeOH (15 mL) was stirred at room temp. for 4 h with NaOMe (1 mL, 0.1 M) at pH 10. The solution was neutralized with Dowex 50 (H^+^) cation-exchange resin and filtered. The filtrate was concentrated and purified by column chromatography (toluene/EtOAc, 1:3) to give **29** (101 mg, 30 %) as the fastest migrating compound. [*α*]_D_^20^ = +78 (*c* = 1.3, CHCl_3_). ^1^H NMR (400 MHz, CDCl_3_): *δ* = 7.41–7.27 (m, 10 H, Ph-H), 5.82 (m, 1 H, –C*H*=), 5.24 [dq, 1 H, =C*H*_2_(*trans*)], 5.16 [dq, 1 H, =C*H*_2_(*cis*)], 4.93 (d, 1 H, C*H*_2_Ph), 4.90 (d, *J*_1′,2′_ = 3.2 Hz, 1 H, 1′-H), 4.81–4.66 (m, 3 H, 1.5 × C*H*_2_Ph), 4.02–3.76 (m, 15 H, 3-H, 4-H, 5-H, 7-H, 8a-H, 8b-H, 2′-H, 3′-H, 4′-H, 5′a-H, CO_2_C*H*_3_, OC*H*_2_), 3.70 (dd, *J*_5′a,5′b_ = 12.3, *J*_5′a,4′_ = 1.7 Hz, 1 H, 5′b-H) and 3.64 (dd, *J*_6,7_ = 8.5, *J*_6,5_ = 1.4 Hz, 1 H, 6-H) ppm. ^13^C NMR (100 MHz, CHCl_3_): *δ* = 167.44 (C-1), 137.16 and 136.47 (C-Ar), 132.87 (–*C*H=), 128.84–127.78 (C-Ar), 117.48 (=*C*H_2_), 101.08 (C-2), 100.68 (C-1′), 81.61 (C-7), 76.83 (C-3′), 75.36 (C-2′), 75.32 and 72.20 (*C*H_2_Ph), 72.14 (C-3), 70.59 (C-6), 67.91 (C-5), 65.45 (C-4), 64.89 (O*C*H_2_), 63.44 (C-8), 61.67 (C-5′), 59.25 (C-4′) and 52.58 (CO_2_CH_3_) ppm.

Further elution of the column with EtOAc/EtOH (2:1) gave **26** as a colorless solid (230 mg, 68 %). [*α*]_D_^20^ = +71.6 (*c* = 0.4, CHCl_3_): ^1^H NMR (600 MHz, CDCl_3_): *δ* = 7.39–7.27 (m, 10 H, Ph-H), 5.79 (m, 1 H, –C*H*=), 5.22 [dq, 1 H, =C*H*_2_(*trans*)], 5.11 [dq, 1 H, =C*H*_2_(*cis*)], 4.99 (d, *J*_1′,2′_ = 3.5 Hz, 1 H, 1′-H), 4.79–4.69 (m, 2 H, 2 × C*H*_2_Ph), 4.08 (dd, 1 H, 5-H), 4.07 (m, 1 H, 7-H), 4.02 (m, 1 H, OC*H*_2_), 3.97 (d, 1 H, 3-H), 3.96 (dd, *J*_3′,2′_ = 10.0, *J*_3′,4′_ = 3.8 Hz, 1 H, 3′-H), 3.88 (dt, *J*_4′,5′a_ = 1.8 Hz, 1 H, 4′-H), 3.86 (dd, 1 H, 2′-H), 3.86 (m, 1 H, 8a-H), 3.82 (dd, *J*_5′a,5′b_ = 12.4 Hz, 1 H, 5′a-H), 3.79 (s, 3 H, CO_2_C*H*_3_), 3.76 (dd, *J*_8b,8a_ = 11.4 Hz, 1 H, 8b-H), 3.76–3.72 (m, 2 H, 4-H, OC*H*_2_), 3.59 (dd, 1 H, 5′b-H) and 3.58 (dd, *J*_6,7_ = 8.2 Hz, 1 H, 6-H) ppm. ^13^C NMR (100 MHz, CHCl_3_): *δ* = 169.49 (C-1), 138.14 and 138.00 (C-Ar), 133.17 (–*C*H=), 128.64, 128.57, 128.14, 128.04 and 127.74 (C-Ar), 117.80 (=*C*H_2_), 101.92 (C-2), 99.03 (C-1′), 77.29 (C-3′), 76.24 (C-2′), 73.82 and 72.88 (*C*H_2_Ph), 71.87 (C-6), 70.32 (C-3), 68.98 (C-8), 68.33 (C-7), 67.25 (C-5), 66.18 (C-4), 65.45 (O*C*H_2_), 60.75 (C-5′), 59.96 (C-4′) and 53.31 (CO_2_CH_3_) ppm. HRMS (ESI-TOF): calcd. for [M + NH_4_]^+^ 663.2872; found 663.2875.

**Acetylation of 26:** A solution of **26** (230 mg, 0.35 mmol) in dry pyridine (3 mL) was stirred with acetic anhydride (2 mL) in pyridine (1 mL) with a catalytic amount of DMAP for 24 h at room temp. The solution was cooled to 0 °C, methanol (3 mL) was added, and stirring was continued for 30 min. The solution was coevaporated with toluene. The residue was directly applied to column chromatography (toluene/EtOAc, 5:1) to give **25** (273 mg, 94 %) as a colorless syrup. [*α*]_D_^20^ = +31 (*c* = 1.7, CHCl_3_). ^1^H NMR (400 MHz, CDCl_3_): *δ* = 7.40–7.27 (m, 10 H, Ph-H), 5.76 (m, 1 H,–C*H*=), 5.50 (dd, *J*_3,4_ = 3.6, *J*_3,5_ = 1.0 Hz, 1 H, 3-H), 5.33 (ddd, *J*_7,6_ = 9.9, *J*_7,8a_ = 3.3, *J*_7,8b_ = 1.9 Hz, 1 H, 7-H), 5.30–5.26 (m, 2 H, 4-H, 5-H), 5.23 [dq, 1 H, =C*H*_2_(*trans*)], 5.10 [dq, 1 H, =C*H*_2_(*cis*)], 4.97 (d, *J*_1′,2′_ = 2.9 Hz, 1 H, 1′-H), 4.78–4.63 (m, 4 H, 2 × C*H*_2_Ph), 4.31 (dd, *J*_6,5_ = 1.8 Hz, 6-H), 4.21 (dq, 1 H, OC*H*_2_), 3.98 (dd, *J*_8a,8b_ = 12.8 Hz, 1 H, 8a-H), 3.90 (dd, 1 H, 8b-H), 3.86–3.78 (m, 4 H, 2′-H, 3′-H, 4′-H, OC*H*_2_), 3.73 (dd, *J*_5′a,5′b_ = 12.3 Hz, 1 H, 5′a-H), 3.73 (s, 3 H, CO_2_C*H*_3_), 3.58 (dd, 1 H, 5′b-H), 2.04 (s, 3 H), 2.03 (s, 3 H), 1.97 (s, 3 H) and 1.93 (s, 3 H, 4 × C*H*_3_CO) ppm. ^13^C NMR (100 MHz, CDCl_3_): *δ* = 170.31, 169.44, 169.20 and 168.93 (4 × O*C*CH_3_), 165.94 (C-1), 138.13, 137.86 (2 × Ph-C*q*), 132.84(–*C*H=), 128.53, 128.48, 127.98, 127.87, 127.79, 127.60 (C-Ar), 118.09 (=*C*H_2_), 99.04 (C-2), 98.33 (C-1′), 76.69 (C-2′), 75.99 (C-3′), 73.71 and 73.07 (2 × *C*H_2_Ph), 69.57 (C-7), 67.80 (C-6), 67.52 (C-3), 66.03 (C-4), 65.41 (O*C*H_2_), 64.97 (C-8), 63.87 (C-5), 60.75 (C-5′), 59.69 (C-4′), 52.62 (CO_2_CH_3_), 20.79, 20.67, 20.52 and 20.48 (4 × OC*C*H_3_) ppm. HRMS (ESI-TOF): calcd. for C_37_H_45_N_3_O_14_ [M + HCOO^–^]^–^ 858.2938; found 858.2953.

**Methyl 4-Azido-2,3-di-*O*-acetyl-4-deoxy-β-l-arabinopyranosyl-(1→8)-(allyl 3,4,5,7-tetra-*O*-acetyl-d-*glycero*-α-d-*talo*-oct-2-ulopyranoside)onate (30):** A solution of **25** (169 mg, 0.207 mmol) in dry chloroform (10 mL) was stripped with argon. A solution of TiCl_4_ (100 μL, 0.913 mmol) in chloroform (2 mL) was added slowly at 0 °C. After 1 h, additional TiCl_4_ (100 μL) was added until TLC showed the complete consumption of starting material to a lower running sport (*R*_f_ = 0.13; EtOAc/toluene, 1:1). Ethyl ether (20 mL) and saturated aqueous NaHCO_3_ solution (10 mL) were added, and the solution was stirred for 30 min. The layers were separated, and the organic phase was dried (MgSO_4_) and concentrated. The residue was purified by flash chromatography on silica gel (toluene/EtOAc, 1:1). The crude product was taken up in pyridine (3 mL), cooled to 0 °C, and a catalytic amount of DMAP and a solution of acetic anhydride (2 mL) in pyridine (1 mL) was added slowly. The solution was stirred for 24 h at room temp. Dry MeOH (3 mL) was added slowly at 0 °C and the solution was stirred for an additional 30 min. The solution was concentrated, coevaporated with toluene five times, and directly subjected to column chromatography. Elution with toluene/EtOAc, 4:1 afforded **30** (133 mg, 0.185 mmol, 89 %) as a colorless syrup. [*α*]_D_^20^ = +48 (*c* = 0.57, CHCl_3_). ^1^H NMR (600 MHz, CDCl_3_): *δ* = 5.89 (m, 1 H, –C*H*=), 5.54 (dd, 1 H, *J*_3,5_ = 0.85 Hz, *J*_3,4_ = 3.7 Hz, 3-H), 5.38 (t, 1 H, *J*_4,5_ = 3.7 Hz, 4-H), 5.33–5.28 [m, 3 H, 5-H, 7-H, =C*H*_2_(*trans*)], 5.23 (dd, 1 H, *J*_3′,4′_ = 3.9, *J*_2′,3′_ = 10.4 Hz, 2′-H), 5.21 [dq, 1 H, =C*H*_2_(*cis*)], 5.15 (dd, 1 H, *J*_3′,2′_ = 10.3, *J*_3′,4′_ = 3.6 Hz, 3′-H), 5.13 (d, 1 H, *J*_1′,2′_ = 3.7 Hz, 1′-H), 4.26 (dd, 1 H, *J*_6,5_ = 1.9, *J*_6,7_ = 9.8 Hz, 6-H), 4.23–4.19 (ddt, 1 H, OC*H*_2_), 4.08 (dt, 1 H, *J*_4′,3′_ = 3.6, *J*_4′,5′a_ = 1.8 Hz, 4′-H), 4.04 (dd, 1 H, *J*_8a,8b_ 13.0 Hz, 8a-H), 3.93–3.89 (ddt, 1 H, OC*H*_2_), 3.90 (dd, 1 H, *J*_5′a,5′b_ = 12.7 Hz, 5′a-H), 3.85 (dd, 1 H, *J*_8b,7_ = 2.2 Hz, 8b-H), 3.75 (s, 3 H, CO_2_CH_3_), 3.67 (dd, 1 H, 5′b-H), 2.12 (s, 3 H), 2.11 (s, 3 H), 2.04 (s, 6 H), 1.99 (s, 3 H) and 1.96 (s, 3 H, 6 × COCH_3_) ppm. ^13^C NMR (150 MHz, CDCl_3_): *δ* = 132.25 (–*C*H=), 117.49 (=*C*H_2_), 99.22 (C-2), 97.36 (C-1′), 68.99 (d.i., C-2′, C-7), 68.21 (C-3′), 67.80 (C-6), 67.55 (C-3), 66.11 (C-4), 65.66 (C-8), 65.17 (O*C*H_2_), 63.77 (C-5), 60.11 (C-5′), 59.60 (C-4′), 52.72 (CO_2_CH_3_), 20.79, 20.61, 20.51, 20.49, 20.49 (6 × OC*C*H_3_) ppm. HRMS (ESI-TOF): calcd. for C_29_H_39_N_3_O_18_ [M + HCOO^–^]^–^ 762.2211; found 762.2224.

**Sodium 4-Azido-4-deoxy-β-l-arabinopyranosyl-(1→8)-(allyl d-*glycero*-α-d-*talo*-oct-2-ulopyranoside)onate (31):** A solution of **30** (10 mg, 0.013 mmol) in dry MeOH (1 mL) was adjusted to pH 10 with NaOMe (0.1 mL, 0.1 M) and stirred for 4 h at room temp. The solution was neutralized with Dowex 50 (H^+^) cation-exchange resin. The suspension was filtered, and the filtrate was concentrated. A solution of the residue in water (1 mL) was stirred with aqueous NaOH (2 mL, 0.2 M) at pH 12 for 3 h at room temp. The solution was neutralized with Dowex 50 (H^+^) cation-exchange resin. The resin was removed by filtration, and the filtrate was lyophilized to give **31** as a white solid (6.3 mg, 97 %). [*α*]_D_^20^ = +102.7 (*c* = 0.4, MeOH). ^1^H NMR (600 MHz, D_2_O): *δ* = 5.84 (m, 1 H, –C*H*=), 5.26–5.21 [dq, 1 H, =C*H*_2_(*trans*)], 5.15–5.12 [dq, 1 H, =C*H*_2_(*cis*)], 4.89 (d, *J*_1′,2′_ = 3.7 Hz, 1 H, 1′-H), 4.09 (ddd, *J*_7,6_ = 8.8 Hz, 1 H, 7-H), 4.01–3.97 (m, 2 H, 3′-H, 5-H), 3.92–3.88 (m, 4 H, 3-H, 4-H, 4′-H, OC*H*_2_), 3.85–3.82 (m, 2 H, 5′a-H, 8a-H), 3.75–3.70 (m, 3 H, 2′-H, 8b-H, OC*H*_2_), 3.65 (dd, *J*_5′b,5′a_ = 12.9 Hz, 1 H, 5′b-H) and 3.58 (dd, 1 H, 6-H) ppm. ^13^C NMR (150 MHz, D_2_O): *δ* = 174.04 (C-1), 134.25 (–*C*H=), 118.29 (=*C*H_2_), 102.66 (C-2), 101.33 (C-1′), 72.41 (C-3), 72.14 (C-6), 70.58 (C-8), 69.54 (C-2′), 69.40 (C3′), 68.85 (C-5), 68.50 (C-7), 67.09 (C-4), 65.03 (O*C*H_2_), 63.08 (C-4′) and 61.21 (C-5′) ppm. HRMS (ESI-TOF): calcd. for C_16_H_25_N_3_O_16_ [M – H]^–^ 450.1365; found 450.1366.

**Ammonium 4-Amino-4-deoxy-β-l-arabinopyranosyl-(1→8)-(allyl d-*glycero*-α-d-*talo*-oct-2-ulopyranoside)onate (32):** A solution of **31** (20 mg, 0.045 mmol) in H_2_O/diisopropylamine (3:1, 2 mL) was stripped with argon in a light protected flask. Dithiothreitol (29.8 mg, 0.183 mmol) was added, and the solution was stirred for 2 h at room temp. under argon. Another portion of dithiothreitol (29.8 mg) was added to complete the conversion. The solution was diluted with H_2_O (2 mL) and washed with chloroform (2 mL) five times. The organic phases were reextracted with water and the combined aqueous phases were stripped with argon until the solution became clear again. The material obtained upon lyophilization was dissolved in H_2_O (1 mL) and transferred to a packed column of Dowex 50 (H^+^) cation-exchange resin. The column was washed with water (20 mL) under TLC control. The product was then eluted with ammonia (20 mL, 1 M) and lyophilized to give **32** (12 mg, 60 %) as a white amorphous solid. [*α*]_D_^20^ = +83 (*c* = 0.29, H_2_O). ^1^H NMR (400 MHz, D_2_O at pD 7.5): *δ* = 5.93 (m, 1 H,–C*H*=), 5.35–5.28 [dq, 1 H, =C*H*_2_(*trans*)], 5.23–5.18 [dq, 1 H, =C*H*_2_(*cis*)], 4.99 (d, *J*_1′,2′_ = ca. 3.7 Hz, 1 H, 1′-H), 4.11–4.03 (m, 3 H, 3′-H, 4-H, 7-H), 4.01 (br. s, 1 H, 5-H), 3.99 (dd, *J*_5′a,5′b_ = 11.5, *J*_5′a,4′_ = 2.0 Hz, 1 H, 5′a-H), 3.94–3.78 (m, 4 H, 8a-H, 8b-H, OC*H*_2_), 3.74 (dd, *J*_2′,1′_ = 3.7, *J*_2′,3′_ = 9.9 Hz, 1 H, 2′-H), 3.68 (dd, 1 H, 5′b-H), 3.61 (dd, *J*_6,5_ = 0.9, *J*_6,7_ = 9.3 Hz, 1 H, 6-H), 3.49 (br. s, 1 H, 4′-H), 2.03 (dd, *J*_3*e*,3*a*_ = 13.0 Hz, 1 H, 3*e*-H), 1.77 (t, 1 H, 3*a*-H) ppm. ^13^C NMR (100 MHz, D_2_O): *δ* = 176.00 (C-1), 134.60 (–*C*H=), 118.50 (=*C*H_2_), 100.80 (C-2), 99.92 (C-1′), 72.33 (C-6), 70.84 (C-8), 68.90, 68.53 (C-2′, C-3′), 67.23 (C-7), 67.03 (C-4), 66.79 (C-5), 65.19 (O*C*H_2_), 60.07 (C-5′), 52.30 (C-4′), 34.92 (C-3) ppm. HRMS (ESI-TOF): calcd. for C_16_H_27_NO_11_ [M + H]^+^ 410.1657; found 410.1659.

**Ammonium 4-Amino-4-deoxy-β-l-arabinopyranosyl-(1→8)-[3-(3-mercaptopropylthio)propyl d-*glycero*-α-d-*talo*-oct-2-ulopyranoside]onate (33):** Propane-1,3-dithiol (0.1 mL) was added to a solution of **32** (2.5 mg, 5.8 μmol) in MeOH (2 mL) and stripped with argon in a quartz flask. The solution was stirred under UV radiation (254 nm) for 1 h. The reaction mixture was diluted with H_2_O (2 mL) and washed with chloroform (2 mL) five times. The organic phases were reextracted with water and the combined aqueous phases were stripped with argon until the solution become clear. The aqueous phase was lyophilized, and the residue was purified on BioGel P-2 to give **33** (2.5 mg, 80 %) as a colorless solid. [*α*]_D_^20^ = +46 (*c* = 0.2, H_2_O). ^1^H NMR (400 MHz, D_2_O): *δ* = 5.06 (d, *J*_1′,2′_ = 3.6 Hz, 1 H, 1′-H), 4.26 (ddd, *J*_8b,7_ = 7.3, *J*_8a,7_ = 2.8, *J*_6,7_ = 8.8 Hz, 1 H, 7-H), 4.13–4.05 (m, 2 H, 5-H, 5′a-H), 4.08 (dd, *J*_2′,3′_ = 9.9, *J*_3′,4′_ = 5.3 Hz, 1 H, 3′-H), 4.01–3.95 (m, 3 H, 3-H, 4-H, 8a-H), 3.92 (dd, *J*_8b,8a_ = 10.6 Hz, 1 H, 8b-H), 3.82 (dd, 1 H, 2′-H), 3.70 (dd, *J*_5′b,5′a_ = 12.8, *J*_5′b,4′b_ = 2.3 Hz, 1 H, 5′b-H), 3.64 (dd, 1 H, 6-H), 3.60–3.53 (m, 1 H, OC*H*_2_), 3.46 (br. m, 1 H, 4′-H), 3.38–3.31 (m, 1 H, OC*H*_2_), 2.73–2.62 (m, 6 H, 3 × CH_2_) and 1.95–1.83 (m, 4 H, 2 × CH_2_) ppm. ^13^C NMR (100 MHz, D_2_O): *δ* = 175.07 (C-1), 102.83 (C-2), 99.86 (C-1′), 72.73 (C-6), 72.71 (C-3), 71.16 (C-8), 69.09 (C-2′, C-5), 68.72 (C-3′), 68.24 (C-7′), 67.34 (C-4), 62.69 (O*C*H_2_), 61.26 (C-5′), 51.23 (C-4′), 33.54 (OCH_2_*C*H_2_CH_2_S), 30.42 (OCH_2_CH_2_*C*H_2_S), 29.46 (S*C*H_2_CH_2_CH_2_SH), 28.83 (SCH_2_*C*H_2_CH_2_SH) and 23.59 (SCH_2_CH_2_*C*H_2_SH) ppm. HRMS (ESI-TOF): calcd. for C_19_H_35_NO_12_S_2_ [M – H]^–^ 532.1528; found 532.1523.

**Dimethyl 4-Azido-2,3-di-*O*-benzyl-4-deoxy-β-l-arabinopyranosyl-(1→8)-[(4,5,7,8-*O*-tetra-*O*-acetyl-3-deoxy-d-*manno*-oct-2-ulopyranosyl)onate-(2→4)]-(allyl 3-deoxy-α-d-*manno*-oct-2-ulopyranoside)onate (35) and Dimethyl 4-Azido-2,3-di-*O*-benzyl-4-deoxy-β-l-arabinopyranosyl-(1→8)-[(4,5,7,8-*O*-tetra-*O*-acetyl-3-deoxy-d-*manno*-oct-2-ulopyranosyl)onate-(2→7)]-(allyl 3-deoxy-α-d-*manno*-oct-2-ulopyranoside)onate (38):** A suspension of **17** (88 mg, 0.139 mmol), Hg(CN)_2_ (23.3 mg, 0.092 mmol), HgBr_2_ (16.6 mg, 0.046 mmol), and 4 Å molecular sieves in dry nitromethane (10 mL) was stirred at room temp. for 2 h. A solution of **34** (67.5 mg, 0.14 mmol) in MeNO_2_ (5 mL) was added over 2 h using a syringe pump. After 6 h, additional portions of **34** (34 mg, 0.007 mmol), Hg(CN)_2_ (12.0 mg, 0.046 mmol), and HgBr_2_ (8.3 mg, 0.023 mmol) were added and stirring was continued for 8 h. The suspension was diluted with EtOAc (50 mL) and filtered through a short plug of Celite®. The Celite bed was washed with EtOAc, and the combined filtrates were washed with aqueous KI solution (15 %), saturated aqueous NaHCO_3_ solution, and dried (MgSO_4_). The organic phase was concentrated, and the residue was directly applied to a column of silica gel. Elution with toluene/EtOAc (1:1) first afforded **38** (8 mg, 5.5 %) as a colorless syrup. [*α*]_D_^20^ = +106 (*c* = 0.9, CHCl_3_). ^1^H NMR (400 MHz, CDCl_3_): *δ* = 7.41–7.25 (m, 10 H, Ar-H), 5.86 (m, 1 H, –C*H*=), 5.39–5.36 (br. s, 1 H, 5′-H), 5.30–5.22 [m, 3 H, 4′-H, 7′-H, =C*H*_2_(*trans*)], 5.12 [dq, 1 H, =C*H*_2_(*cis*)], 5.01 (dd, *J*_1″,2″_ = 3.9 Hz, 1 H, 1″-H), 4.86–4.64 (m, 4 H, 2 × OCH_2_Ph), 4.60 (dd, *J*_8′b,8′a_ = 11.5, *J*_8′b,7′_ = 2.8 Hz, 1 H, 8a′-H), 4.33 (dd, *J*_6′,7′_ = 9.4, *J*_6′,5′_ = 1.4 Hz, 1 H, 6′-H), 4.19 (m, 1 H, 7-H), 4.09–3.98 (m, 4 H, 3″-H, 4″-H, 8′b-H, OC*H*_2_), 3.88 (dd, *J*_3″,2″_ = 9.0 Hz, 1 H, 2″-H), 3.86–3.70 (m, 13 H, 4-H, 5-H, 5″a-H, 6-H, 8a-H, 8b-H, 2 × CO_2_C*H*_3_, OC*H*_2_), 3.65 (dd, *J*_5″b,5″a_ = 12.3 Hz, 1 H, 5″b-H), 2.19 (dd, *J*_3*e*,3*a*_ = 12.9, *J*_3e,4_ = 4.9 Hz, 1 H, 3*e*-H), 2.13–1.88 (m, 14 H, 3′*a*-H, 3′*e*-H, 4 × COC*H*_3_) and 1.46 (dd, *J*_4,3*a*_ = 11.8 Hz, 1 H, 3*a*-H) ppm. ^13^C NMR (100 MHz, CDCl_3_): *δ* = 170.80, 170.37, 169.87 and 169.63 (4 × O*C*CH_3_), 168.41 (C-1′), 168.09 (C-1), 137.94 (C-Ar), 137.84 (C-Ar), 133.68 (–*C*H=), 128.42–127.58 (C-Ar), 116.78 (=*C*H_2_), 99.23 (C-2′), 98.82 (C-1″), 98.70 (C-2), 77.72 (C-3″), 75.59 (C-2″), 74.46 (C-7), 74.12 and 72.62 (2 × O*C*H_2_Ph), 73.58 (C-6), 70.07 (C-6′), 67.76 (C-4), 67.57 (C-7′), 66.53 (C-8), 65.93 (C-5), 65.84 (C-4′), 64.64 (O*C*H_2_), 64.23 (C-5′), 63.00 (C-8′), 60.85 (C-5″), 59.52 (C-4″), 53.14 and 52.41 (2 × CO_2_CH_3_), 34.68 (C-3′), 32.31 (C-3), 20.75, 20.70, 20.65 and 20.59 (4 × OC*C*H_3_) ppm.

Continued elution of the column furnished a small amount of the β-(2→4)-linked trisaccharide (2 mg, 1.4 %) followed by **35** (27 mg, 19 %) as a colorless oil. [*α*]_D_^20^ = +55 (*c* = 0.4, CHCl_3_). ^1^H NMR (400 MHz, CDCl_3_): *δ* = 7.42–7.27 (m, 10 H, Ar-H), 5.74 (m, 1 H,–C*H*=), 5.40–5.37 (br. s, 1 H, 5′-H), 5.29 (ddd, *J*_4′,5′_ = 3.0, *J*_4′,3′e_ = 4.7, *J*_4′,3′a_ = 12.2 Hz, 1 H, 4′-H), 5.20 (dt, 1 H, 7′-H), 5.16 [dq, 1 H, =C*H*_2_(*trans*)], 5.06 [dq, 1 H, =C*H*_2_(*cis*)], 4.86–4.65 (m, 6 H, 2 × OCH_2_Ph, 1″-H, 8a′-H), 4.28 (ddd, 1 H, 4-H), 4.17 (dd, *J*_6′,7′_ = 9.4, *J*_6′,5′_ = 1.0 Hz, 1 H, 6′-H), 4.14–4.07 (br. s, 1 H, 7-H), 4.03 (dd, *J*_8′b,8′a_ = 12.4, *J*_8′b,7′_ = 3.6 Hz, 1 H, 8′b-H), 3.98 (dd, *J*_3″,2″_ = 9.6, *J*_3″,4″_ = 3.5 Hz, 1 H, 3″-H), 3.96–3.74 (m, 13 H, OC*H*_2_, 2″-H, 4″-H, 5″a-H, 8a-H, 8b-H, 2 × CO_2_C*H*_3_), 3.59 (dd, *J*_5″b,5″a_ = 12.3, *J*_5″b,4″_ = 2.1 Hz, 1 H, 5″b-H), 3.43 (dd, 1 H, 6-H), 2.28 (ddd, *J*_3*e*,3*a*_ = 12.4, *J*_3e,4_ = 4.8 Hz, 1 H, 3*e*-H), 2.16–1.92 (m, 15 H, 3*a*-H, 3′*a*-H, 3′*e*-H, 4 × COC*H*_3_) ppm. ^13^C NMR (100 MHz, CDCl_3_): *δ* = 170.77, 170.28, 169.91 and 169.63 (4 × O*C*CH_3_), 168.23 (C-1′), 168.04 (C-1), 137.72 (C-Ar), 137.64 (C-Ar), 133.61 (–*C*H=), 129.00–125.27 (C-Ar), 116.81 (=*C*H_2_), 100.11 (C-1″), 98.50, 97.68 (C-2, C-2′), 77.10 (C-3″), 76.08 (C-2″), 74.41, 72.63 (2 × O*C*H_2_Ph), 71.35 (C-6), 71.32 (C-8), 69.26 (C-6′), 68.67 (C-4), 67.80 (C-7′), 67.44 (C-7), 66.11 (C-4′), 64.28 (C-5′), 64.18 (O*C*H_2_), 63.55 (C-5), 61.33 (C-8′), 60.93 (C-5″), 59.77 (C-4″), 53.04, 52.50 (2 × CO_2_CH_3_), 32.98 (C-3′), 32.25 (C-3), 20.70, 20.70, 20.61 and 20.59 (4 × OC*C*H_3_) ppm. HRMS (ESI-TOF): calcd. for C_48_H_61_N_3_O_22_ [M + Na]^+^ 1054.3639; found 1054.3641.

**Dimethyl 4-Azido-2,3-di-*O*-benzyl-4-deoxy-β-l-arabinopyranosyl-(1→8)-[(4,5,7,8-*O*-tetra-*O*-acetyl-3-deoxy-α-d-*manno*-oct-2-ulopyranosyl)onate-(2→4)](allyl 5,7-di-*O*-acetyl-3-deoxy-α-d-*manno*-oct-2-ulopyranoside)onate (36):** Acetic anhydride (0.2 mL) was added to a solution of **35** (36 mg, 0.034 mmol) in dry pyridine (4 mL) and a catalytic amount of DMAP at 0 °C. The solution was stirred for 12 h at room temp. and the reaction was quenched by the addition of dry MeOH (1 mL) at 0 °C. The solution was concentrated and coevaporated several times with toluene. The residue was dissolved in CHCl_3_, washed with saturated aqueous NaHCO_3_ solution and dried (MgSO_4_). The solution was concentrated and the residue was purified by column chromatography on silica gel (toluene/EtOAc, 1:1) to afford **36** (29 mg, 75 %) as a colorless syrup. [*α*]_D_^20^ = +63.5 (*c* = 0.2, CHCl_3_). ^1^H NMR (400 MHz, CDCl_3_): *δ* = 7.36–7.26 (m, 10 H, Ar-H), 5.75 (m, 1 H, –C*H*=), 5.35 (br. s, 1 H, 5′-H), 5.22 (br. s, 1 H 5-H), 5.21–55.12 [m, 4 H, 4′-H, 7′-H, 7-H, =C*H*_2_(*trans*)], 5.05 [dq, 1 H, =C*H*_2_(*cis*)], 4.86 (dd, *J*_1″,2″_ = 3.3 Hz, 1 H, 1″-H), 4.78 (dd, *J*_8′b,8′a_ = 12.3, *J*_8′a,7′_ = 2.6 Hz, 1 H, 8a′-H), 4.75–4.64 (m, 5 H, 2 × OCH_2_Ph, 4-H), 4.14 (dd, *J*_6′,7′_ = 9.4, *J*_6′,5′_ = 1.5 Hz, 1 H, 6′-H), 4.08 (m, 1 H, OC*H*_2_), 4.05 (dd, *J*_6,7_ = 9.7, *J*_6,5_ = 1.2 Hz, 1 H, 6-H), 4.01 (dd, *J*_8′b,7′_ = 3.8 Hz, 1 H, 8′b-H), 3.89 (dd, *J*_3″,2″_ = 9.1, *J*_3″,4″_ = 3.6 Hz, 1 H, 3″-H), 3.88–3.78 (m, 10 H, 2″-H, 4″-H, 8a-H, 8b-H, 2 × CO_2_C*H*_3_), 3.71 (dd, *J*_5″b,5″a_ = 12.3, *J*_5″b,4″_ = 2.0 Hz, 1 H, 5″a-H), 3.56 (dd, *J*_5″b,4″_ = 3.0 Hz, 1 H, 5″b-H), 2.13 (ddd, *J*_3*e*,3*a*_ = 12.6, *J*_3*e*,4_ = 5.1 Hz, 1 H, 3*e*-H), 2.11 (t, *J*_3′a,4′_ = *J*_3′a,3′e_ = 12.7 Hz, 1 H, 3′*a*-H), 2.08–1.92 (m, 20 H, 3*a*-H, 3′*e*-H, 6 × COC*H*_3_) ppm. ^13^C NMR (100 MHz, CDCl_3_): *δ* = 170.68, 170.43, 170.33, 170.10, 169.65 and 169.54 (6 × O*C*CH_3_), 167.85 and 167.04 (C-1′, C-1), 138.35 (C-Ar), 137.98 (C-Ar), 133.58 (–*C*H=), 128.45, 128.42, 127.90, 127.81, 127.76 and 127.68 (C-Ar), 117.29 (=*C*H_2_), 98.59 and 97.62 (C-2, C-2′), 98.52 (C-1″), 76.93 (C-3″), 75.80 (C-2″), 73.39, 73.12 (2 × O*C*H_2_Ph), 69.37 (C-6′), 69.29 (C-6), 68.84 (C-7), 67.92 (C-7′), 66.56 (C-4), 66.35 (C-4′), 66.10 (C-8), 65.12 (C-5), 64.83 (O*C*H_2_), 64.35 (C-5′), 61.35 (C-8′), 60.85 (C-5″), 59.73 (C-4″), 52.73 and 52.56 (2 × CO_2_CH_3_), 34.04 (C-3), 31.57 (C-3′), 20.82–20.54 (6 × OC*C*H_3_) ppm. HRMS (ESI-TOF): calcd. for C_53_H_66_N_3_O_26_ [M + HCOO^–^]^–^ 1160.3940; found 1160.3958.

**Dimethyl 4-Azido-2,3-di-*O*-acetyl-4-deoxy-β-l-arabinopyranosyl-(1→8)-[(4,5,7,8-*O*-tetra-*O*-acetyl-3-deoxy-α-d-*manno*-oct-2-ulopyranosyl)onate-(2→4)]-(allyl 5,7-di-*O*-acetyl-3-deoxy-α-d-*manno*-oct-2-ulopyranoside)onate (39):** A solution of **36** (27 mg, 0.024 mmol) in chloroform (3 mL) was stripped with argon. A solution of TiCl_4_ (5.8 μL, 0.053 mmol) in chloroform (1 mL) was added slowly at 0 °C. After stirring for 12 h at room temp., additional TiCl_4_ (2.9 μL, 0.026 mmol) was added until TLC showed the complete consumption of starting material to a lower migrating spot. Ethyl ether (5 mL) and saturated aqueous NaHCO_3_ solution (5 mL) were added and the solution was stirred for 30 min at room temp. The layers were separated and the organic phase was dried (MgSO_4_) and concentrated. The crude product was taken up in pyridine (3 mL), and a catalytic amount of DMAP and acetic anhydride (0.3 mL) were added slowly. The solution was stirred for 24 h at room temp. and processed as described for **36**. The residue was purified by column chromatography (toluene/EtOAc, 50:1) to afford **39** (17 mg, 68 %) as a colorless syrup. [*α*]_D_^20^ = +96 (*c* = 0.4, CHCl_3_). ^1^H NMR (600 MHz, CDCl_3_): *δ* = 5.88 (m, 1 H, –C*H*=), 5.35–5.33 (br. s, 1 H, 5′-H), 5.25 [dq, 1 H, =C*H*_2_(*trans*)], 5.21–5.16 (m, 2 H, 3″-H, 4′-H), 5.15–5.10 [m, 5 H, 1″-H, 2″-H, 5-H, 7′-H, =C*H*_2_(*cis*)], 5.01 (dt, *J*_7,6_ = 9.8, *J*_7,8a_ = 2.3 Hz, 1 H, 7-H), 4.80 (dd, *J*_8′a,7′_ = 2.4, *J*_8′a,8′b_ = 12.2 Hz, 1 H, 8′a-H), 4.72 (ddd, *J*_4,5_ = 3.0, *J*_4,3e_ = 4.8, *J*_4,3a_ = 11.9 Hz, 1 H, 4-H), 4.15 (dq, 1 H, OC*H*_2_), 4.13 (dd, *J*_6′,5′_ = 1.1, *J*_6′,7′_ = 9.4 Hz, 1 H, 6′-H), 4.11–4.08 (br. s, 1 H, 4″-H), 4.05 (dd, *J*_6,5_ = 1.1, *J*_6,7_ = 9.9 Hz, 1 H, 6-H), 3.97 (dd, *J*_8′b,7′_ = 3.9, *J*_8′b,8′a_ = 12.3 Hz, 1 H, 8′b-H), 3.93 (dd, *J*_8a,7_ = 2.8, *J*_8a,8b_ = 13.1 Hz, 1 H, 8a-H), 3.92 (dq, 1 H, OC*H*_2_), 3.88 (dd, *J*_5″a,4″_ = 0.6, *J*_5″a,5″b_ = 12.2 Hz, 1 H, 5″a-H), 3.82 and 3.81 (2 × CO_2_C*H*_3_), 3.79 (dd, *J*_8b,7_ = 1.8 Hz, 1 H, 8b-H), 3.64 (dd, *J*_5″b,4″_ = 1.9 Hz, 1 H, 5″b-H), 2.21–1.94 (m, 28 H, 3*e*-H, 3*e*′-H, 3*a*-H, 3*a*′-H, 8 × COC*H*_3_) ppm. ^13^C NMR (150 MHz, CDCl_3_): *δ* = 170.69, 170.44, 170.42, 170.31, 170.13, 170.11, 169.64 and 169.49, (8 × *C*OCH_3_), 167.81 (C-1′), 167.04 (C-1), 133.29 (–*C*H=), 116.84 (=*C*H_2_), 98.61, 97.31 (C-2, C-2′), 97.15 (C-1″), 69.39 (C-6), 69.36 (C-7), 69.12 (C-3″), 68.12 (C-6′), 68.06 (C-7′), 67.85 (C-2″), 66.34 (C-4′), 66.24 (C-4), 65.14 (C-8), 64.95 (C-5), 64.61 (O*C*H_2_), 64.28 (C-5′), 61.33 (C-8′), 59.97 (C-5″), 59.53 (C-4″), 52.66 and 52.63 (2 × CO_2_*C*H_3_), 34.11 (C-3), 31.39 (C-3′), 20.82–20.54 (8 × CO*C*H_3_) ppm. HRMS (ESI-TOF): calcd. for C_43_H_58_N_3_O_28_ [M + HCOO^–^]^–^ 1064.3212; found 1064.3205.

**Disodium 4-Azido-4-deoxy-β-l-arabinopyranosyl-(1→8)-[(3-deoxy-α-d-*manno*-oct-2-ulopyranosyl)onate-(2→4)]-(allyl 3-deoxy-α-d-*manno*-oct-2-ulopyranoside)onate (40):** A solution of **39** (5 mg, 0.004 mmol) in dry methanol (1 mL) was stirred with NaOMe (0.1 mL, 0.1 M) for 4 h at room temp. The solution was neutralized with Dowex 50 (H^+^) cation-exchange resin. The resin was removed by filtration, and the filtrate was concentrated. A solution of the residue in water (1 mL) was treated with aqueous NaOH (2 mL, 0.1 M) at room temp. for 3 h. The solution was neutralized with Dowex 50 (H^+^) cation-exchange resin. The resin was removed by filtration, and the filtrate was lyophilized to give **40** (3 mg, 96 %) as a white foamy solid. [*α*]_D_^20^ = +112.6 (*c* = 0.3, H_2_O). ^1^H NMR (600 MHz, D_2_O): *δ* = 5.87 (m, 1 H, –C*H*=), 5.26 [dq, 1 H, =C*H*_2_(*trans*)], 5.15 [dq, 1 H, =C*H*_2_(*cis*)], 4.86 (d, *J*_1″,2″_ = 3.7 Hz, 1 H, 1″-H), 4.08 (ddd, *J*_4,5_ = 2.7, *J*_4,3e_ = 5.0, *J*_4,3a_ = 11.9 Hz, 1 H, 4-H), 4.00–3.94 (m, 4 H, 3″-H, 4′-H, 5-H, 7-H), 3.94–3.92 (br. s, 1 H, 5′-H), 3.91 (dt, *J*_4″,3″_ = 4.0 Hz, 1 H, 4″-H), 3.89–3.85 (m, 2 H, 7′-H, 8′a-H), 3.83 (dq, 1 H, OC*H*_2_)_,_ 3.81 (dd, *J*_5″a,5″b_ = 12.8, *J*_5″a,4″b_ = 1.6 Hz, 1 H, 5″a-H), 3.77 (dd, *J*_8a,8b_ = 10.9, *J*_8a,7_ = 5.6 Hz, 1 H, 8a-H), 3.73 (dq, 1 H, OC*H*_2_), 3.69 (dd, *J*_2″,3″_ = 10.0 Hz, 1 H, 2″-H), 3.66–3.62 (m, 3 H, 5″b-H, 8′b-H, 8b-H), 3.52 (dd, *J*_6,7_ = 9.2 Hz, 1 H, 6-H), 3.51 (dd, *J*_6′,7′_ = 8.5 Hz, 1 H, 6′-H), 2.04 (dd, *J*_3′e,3′a_ = 13.2, *J*_3′e,4′_ = 4.8 Hz, 1 H, 3′*e*-H), 1.90 (dd, *J*_3e,3a_ = 13.1, *J*_3e,4_ = 5.0 Hz, 1 H, 3*e*-H), 1.83 (t, 1 H, 3*a*-H) and 1.67 (t, *J*_3′a,4′_ = 13.1 Hz, 1 H, 3′*a*-H) ppm. ^13^C NMR (150 MHz, D_2_O): *δ* = 176.67 (C-1′), 176.57 (C-1), 134.76 (–*C*H=), 117.82 (=*C*H_2_), 100.89 (C-2′), 100.28 (C-1″), 100.04 (C-2), 73.09 (C-6′), 71.95 (C-6), 70.63 (C-7′), 70.51 (C-8), 69.50 (C-2″), 69.45 (C-3″), 69.23 (C-4), 68.52 (C-7), 66.99 (C-5′), 66.64 (C-4′), 64.92 (C-5), 64.70 (O*C*H_2_), 63.87 (C-8′), 63.09 (C-4″), 61.20 (C-5″), 35.24 (C-3′) and 33.94 (C-3) ppm. HRMS (ESI-TOF): calcd. for C_24_H_36_N_3_O_18_ [M – H]^–^ 654.1999; found 654.2005.

**Disodium 4-Amino-4-deoxy-β-l-arabinopyranosyl-(1→8)-[(3-deoxy-α-d-*manno*-oct-2-ulopyranosyl)onate-(2→4)]-(allyl 3-deoxy-α-d-*manno*-oct-2-ulopyranoside)onate (41):** A solution of **40** (3 mg, 4.5 μmol) in THF/0.1 M NaOH (1:2, 1 mL) was stirred with trimethylphosphane (1.3 μL, 18.3 μmol) for 4 h at room temp. The solution was diluted with H_2_O (2 mL) and washed with chloroform (2 mL) five times. The organic phases were reextracted with water. The combined aqueous phases were stripped with argon until the solution became clear again. The solution was lyophilized, and the residue was purified by gel chromatography (BioGel P-2) and lyophilized to give **41** (2.8 mg, 99 %) as a colorless amorphous solid. [*α*]_D_^20^ = +46.5 (*c* = 0.4, H_2_O). ^1^H NMR (600 MHz, D_2_O): *δ* = 5.93 (m, 1 H, –C*H*=), 5.32 [dq, 1 H, =C*H*_2_(*trans*)], 5.22 [dq, 1 H, =C*H*_2_(*cis*)], 4.98 (d, *J*_1″,2″_ = 3.7 Hz, 1 H, 1″-H), 4.22 (ddd, *J*_4,5_ = 2.6, *J*_4,3e_ = 5.2, *J*_4,3a_ = 12.0 Hz, 1 H, 4-H), 4.10 (dd, *J*_3″,2″_ = 9.9, *J*_3″,4″_ = 4.7 Hz, 1 H, 3″-H), 4.07–4.02 (m, 3 H, 4′-H, 5-H, 7-H), 4.00 (br. s, 1 H, 5′-H), 3.94–3.90 (m, 3 H, 7′-H, 8′a-H, OC*H*_2_), 3.83 (dq, 1 H, OC*H*_2_)_,_ 3.81 (dd, *J*_8a,8b_ = 10.9, *J*_8a,7_ = 6.0 Hz, 1 H, 8a-H), 3.77 (dd, *J*_8b,7_ = 3.0 Hz, 1 H, 8b-H), 3.72 (dd, *J*_5″b,5″a_ = 12.9, *J*_5″b,4″_ = 1.0 Hz, 1 H, 5″b-H), 3.71 (dd, 1 H, 2″-H), 3.68 (dd, *J*_8′b,8′a_ = 13.0 Hz, 1 H, 8′b-H), 3.62 (br. s, 1 H, 4″-H), 3.59 (dd, *J*_6′,7′_ = 9.8 Hz, 1 H, 6′-H), 3.57 (dd, *J*_6,7_ = 9.5 Hz, 1 H, 6-H), 2.12 (dd, *J*_3′e,3′a_ = 13.1, *J*_3′e,4′_ = 5.1 Hz, 1 H, 3′*e*-H), 2.00 (dd, *J*_3e,3a_ = 13.6 Hz, 1 H, 3*e*-H), 1.95 (dd, *J*_3a,4_ = 12.4 Hz, 1 H, 3*a*-H), 1.78 (dd, 1 H, 3′*a*-H) ppm. ^13^C NMR (150 MHz, D_2_O): *δ* = 175.97 and 175.06 (C-1, C-1′), 134.07 (–*C*H=), 117.13 (=*C*H_2_), 100.20 and 99.32 (C-2′, C-2), 99.15 (C-1″), 72.40 (C-6′), 71.41 (C-6), 70.00 (C-8), 70.63 (C-7′), 68.50 (C-4), 68.17 (C-2″), 67.86 (C-7), 66.27 (C-5′), 66.13 (C-4′), 65.92 (C-3″), 64.20 (C-5), 64.03 (O*C*H_2_), 63.18 (C-8′), 57.97 (C-5″), 51.66 (C-4″), 34.51 (C-3′) and 33.21 (C-3) ppm. HRMS (ESI-TOF): calcd. for C_24_H_39_NO_18_ [M + H]^+^ 630.2240; found 630.2233.

**Disodium 4-Amino-4-deoxy-β-l-arabinopyranosyl-(1→8)-[(3-deoxy-α-d-*manno*-oct-2-ulopyranosyl)onate-(2→4)]-[3-(3-mercaptopropylthio)propyl 3-deoxy-α-d-*manno*-oct-2-ulopyranoside]onate (42):** Propane-1,3-dithiol (50 μL) was added to a solution of **41** (2.5 mg, 6.1 μmol) in MeOH (2 mL) and stripped with argon in a quartz flask. The solution was stirred under UV radiation (254 nm) for 1 h to give full conversion. The reaction mixture was diluted with H_2_O (2 mL) and extracted with chloroform (2 mL) five times. The organic phases were reextracted with water, and the combined aqueous phases were stripped with argon until the solution became clear. The solution was lyophilized, and the residue was desalted on BioGel P-2 (size 28 × 1 cm) to give **42** as a white solid (2.8 mg, 99 %). [*α*]_D_^20^ = +45 (*c* = 0.3, H_2_O). ^1^H NMR (600 MHz, D_2_O): *δ* = 4.98 (br. s, 1 H, 1″-H), 4.16–3.93 (m, 9 H, 3″-H, 4-H, 4′-H, 5-H, 5′-H, 5″a-H, 7-H, 7′-H, 8′a-H), 3.87–3.81 (m, 2 H, 8a-H, 8b-H), 3.80–3.77 (dd, 1 H, 2″-H), 3.72 (dd, 1 H, 8′b-H), 3.64–3.56 (m, 2 H, 5″b-H, 6-H), 3.54–3.51 (m, 1 H, 6′-H), 3.47–3.43 [br. s, 1 H, OC*H*_2_(CH_2_)_2_S], 3.34–3.29 [m, 2 H, 4″-H, OC*H*_2_(CH_2_)_2_S], 2.71–2.59 (m, 6 H, 3 × CH_2_), 2.13 (dd, 1 H, 3′*e*-H), 2.04–1.79 (m, 6 H, 3*e*-H, 3*a*-H, 2 × CH_2_), 1.71 (t, 1 H, 3′*a*-H) ppm. HRMS (ESI-TOF): calcd. for C_27_H_47_NO_18_S_2_ [M + H]^+^ 738.2307; found 738.2304.

**Synthesis of Neoglycoconjugates 43–45:** Thiol **24** (2.1 mg) was dissolved in a freshly prepared conjugation buffer [0.5 mL, 20 mM sodium phosphate buffer, 100 mM ethylenediaminetetraacetic acid (EDTA), 80 mM sucrose, pH 6.6] and immediately added to a solution of 5 mg/mL maleimide-activated BSA (20 mM sodium phosphate buffer, 230 mM NaCl, 2 mM EDTA, 80 mM sucrose, pH 6.6). The reaction mixture was purged with argon for 2 min and stirred at room temp. for 2 h. The reaction mixture was purified over a Sephadex G-25M column (0.01 M phosphate-buffered saline in double distilled H_2_O as eluent). The protein-containing fractions were pooled, lyophilized, and purified again over a P-2 gel column (30 × 1 cm, water) to afford **44** (18.4 mg) as a colorless solid. Neoglycoconjugate **45** was prepared from **33** (2.0 mg) by this procedure. Yield of **45**: 20 mg. Neoglycoconjugate **43** was synthesized from **42** (2.0 mg) accordingly to give **43** (6.4 mg) as a colorless powder. MS (MALDI-TOF) measurements of the BSA-conjugates gave maximum peak intensities at 72733 for **43**, 77264 for **44**, and 75165 Da for **45**, which correspond to average ligand-to-protein ratios of 5.4 for **43**, 16.5 for **44**, and 13.6 mol/mol for **45**.

## Supporting Information

(see footnote on the first page of this article): ^1^H and ^13^C NMR spectra of deprotected oligosaccharides.
