# Supplementary material for: Synthesis of Neoglycoconjugates Containing 4-Amino-4-deoxy-l-arabinose Epitopes Corresponding to the Inner Core of Burkholderia and Proteus Lipopolysaccharides
Source: European J Org Chem. 2011 Nov 16;2012(1):119–31. doi: 10.1002/ejoc.201101171 (PMC3482937; doi:10.1002/ejoc.201101171)

Eur. J. Org. Chem. **2011** · © WILEY-VCH Verlag GmbH & Co. KGaA, 69451 Weinheim, 2011 · ISSN 1434–193X

SUPPORTING INFORMATION

DOI: 10.1002/ejoc.201101171
Title: Synthesis of Neoglycoconjugates Containing 4-Amino-4-deoxy-L-arabinose Epitopes Corresponding to the Inner Core of *Burkholderia* and *Proteus* Lipopolysaccharides
Author(s): Markus Blaukopf, Bernhard Müller, Andreas Hofinger, Paul Kosma*

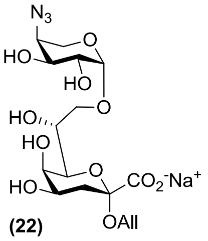
N3
HO
HO
O
O
HO
HO
O
HO
CO2-Na+
OAll

(22)

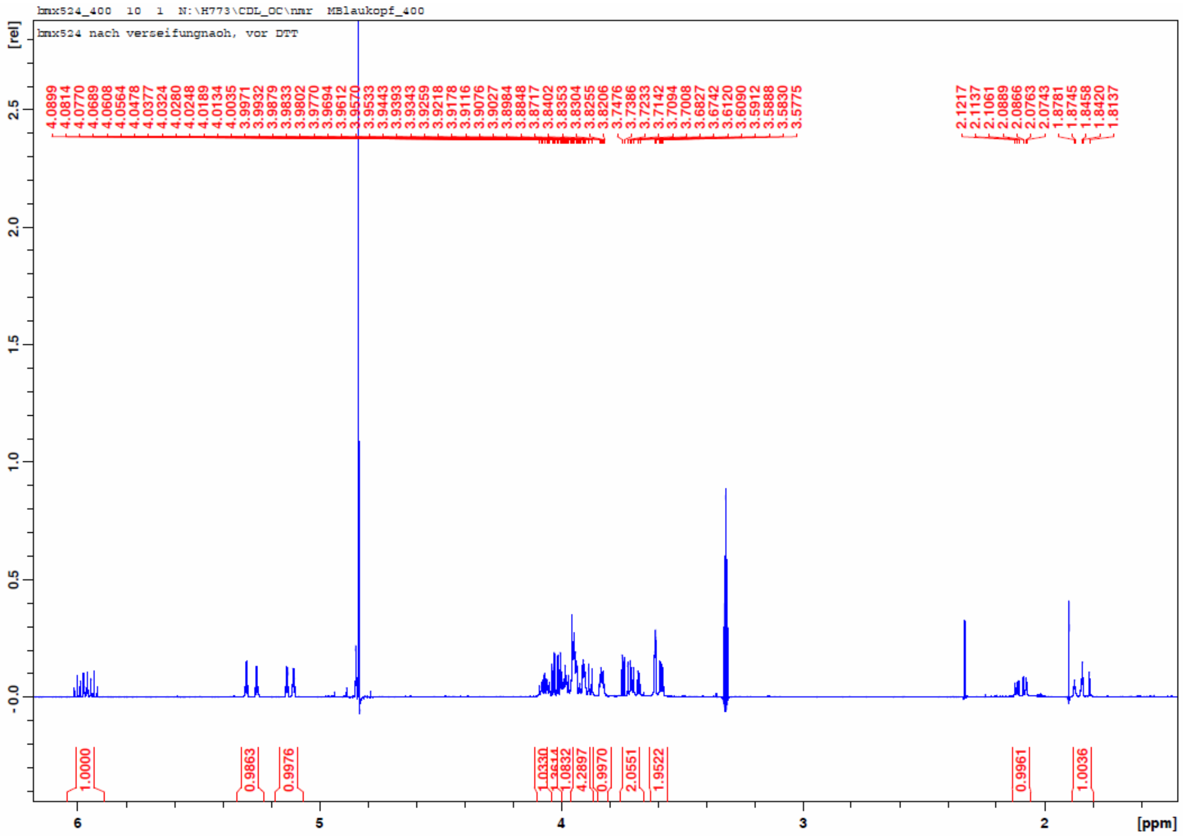
bmx524_400 10 1 N:\H773\CDL_OC\nmr MBlaukopf_400
bmx524 nach verseifungnaoh, vor DTT
[rel]
4.0899
4.0814
4.0770
4.0689
4.0608
4.0564
4.0478
4.0377
4.0324
4.0280
4.0248
4.0189
4.0134
4.0035
3.9971
3.9932
3.9879
3.9833
3.9802
3.9770
3.9694
3.9612
3.9570
3.9533
3.9443
3.9393
3.9343
3.9259
3.9218
3.9178
3.9116
3.9076
3.9027
3.8984
3.8848
3.8717
3.8402
3.8353
3.8304
3.8255
3.8206
3.7476
3.7386
3.7233
3.7142
3.7094
3.7008
3.6827
3.6742
3.6120
3.6090
3.5912
3.5888
3.5830
3.5775
2.1217
2.1137
2.1061
2.0889
2.0866
2.0763
2.0743
1.8781
1.8745
1.8458
1.8420
1.8137
1.0000
0.9863
0.9976
1.0330
[illegible]
1.0832
4.2897
0.9970
2.0551
1.9522
0.9961
1.0036
[ppm]

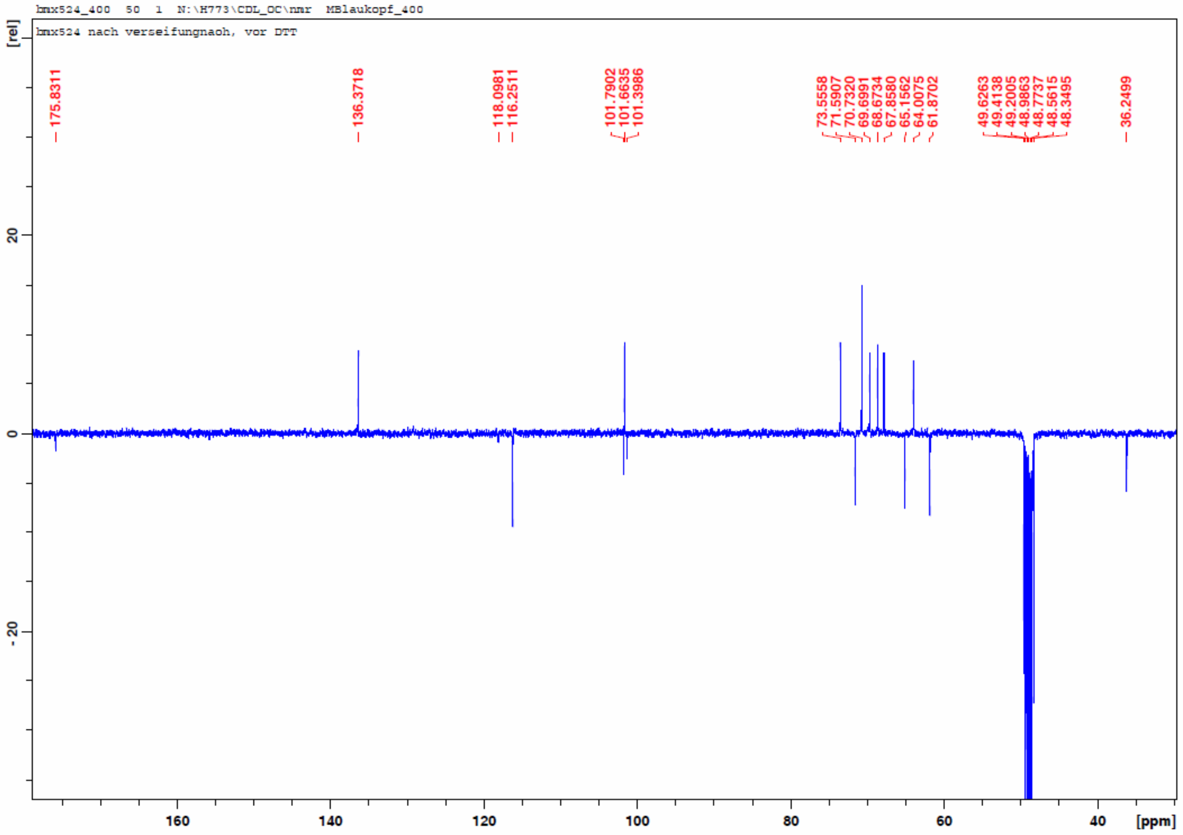
bmx524_400 50 1 N:\H773\CDL_OC\nmr MBlaukopf_400
bmx524 nach verseifungnaoh, vor DTT
[rel]
175.8311
136.3718
118.0981
116.2511
101.7902
101.6635
101.3986
73.5558
71.5907
70.7320
69.6991
68.6734
67.8580
65.1562
64.0075
61.8702
49.6263
49.4138
49.2005
48.9863
48.7737
48.5615
48.3495
36.2499
[ppm]

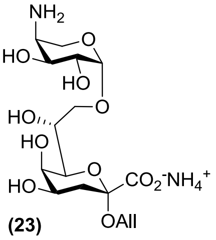
NH2
HO
O
HO
O
HO
HO
O
CO2-NH4+
HO
OAll

(23)

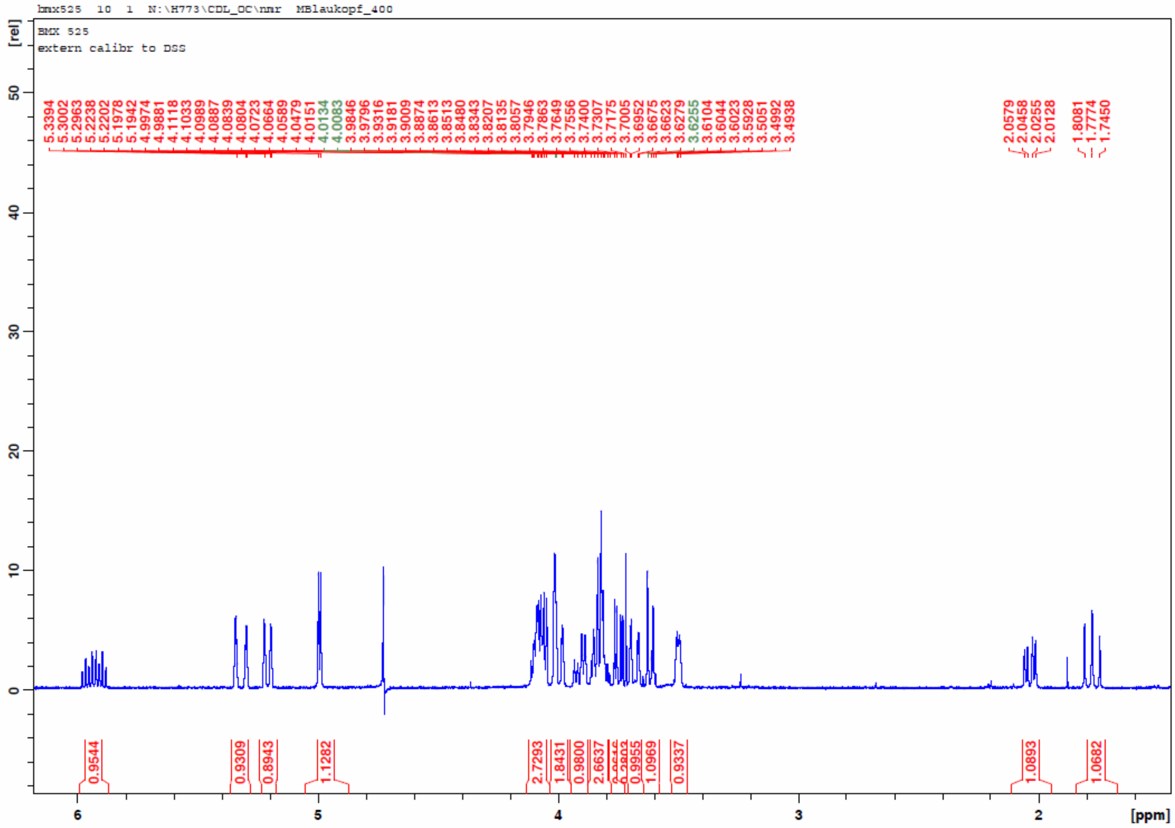
bmx525 10 1 N:\H773\CDL_OC\nmr MBlaukopf_400
BMX 525
extern calibr to DSS
5.3394 5.3002 5.2963 5.2238 5.2202 5.1978 5.1942 4.9974 4.9881 4.1118 4.1033 4.0989 4.0887 4.0839 4.0804 4.0723 4.0664 4.0589 4.0479 4.0151 4.0134 4.0083 3.9846 3.9796 3.9316 3.9181 3.9009 3.8874 3.8613 3.8480 3.8343 3.8207 3.8135 3.8057 3.7946 3.7863 3.7649 3.7556 3.7400 3.7307 3.7175 3.7005 3.6952 3.6675 3.6623 3.6279 3.6255 3.6104 3.6044 3.6023 3.5928 3.5051 3.4992 3.4938
2.0579 2.0458 2.0255 2.0128
1.8081 1.7774 1.7450
0.9544 0.9309 0.8943 1.1282 2.7293 1.8431 0.9800 2.6637 0.9955 1.0969 0.9337 1.0893 1.0682
[rel]
[ppm]

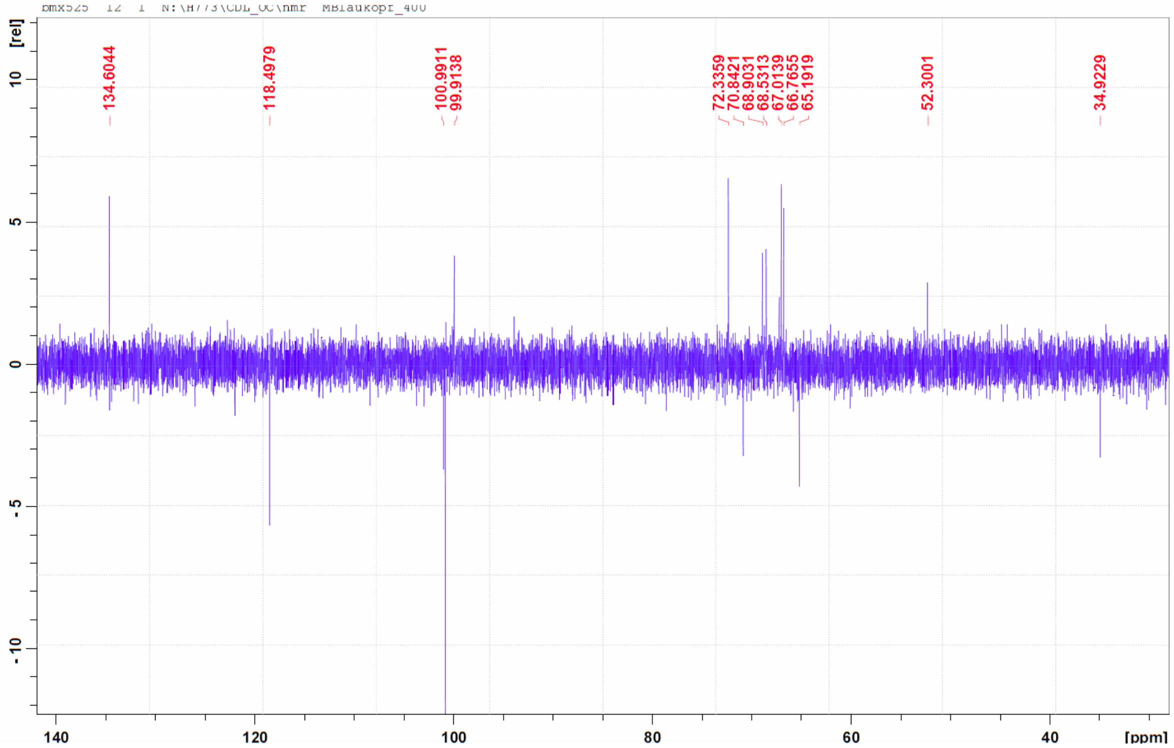
bmx525 12 1 N:\H773\CDL_OC\nmr MBlaukopf_400
134.6044
118.4979
100.9911
99.9138
72.3359
70.8421
68.9031
68.5313
67.0139
66.7655
65.1919
52.3001
34.9229
[rel]
[ppm]

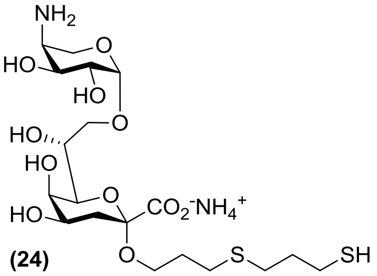

(24)

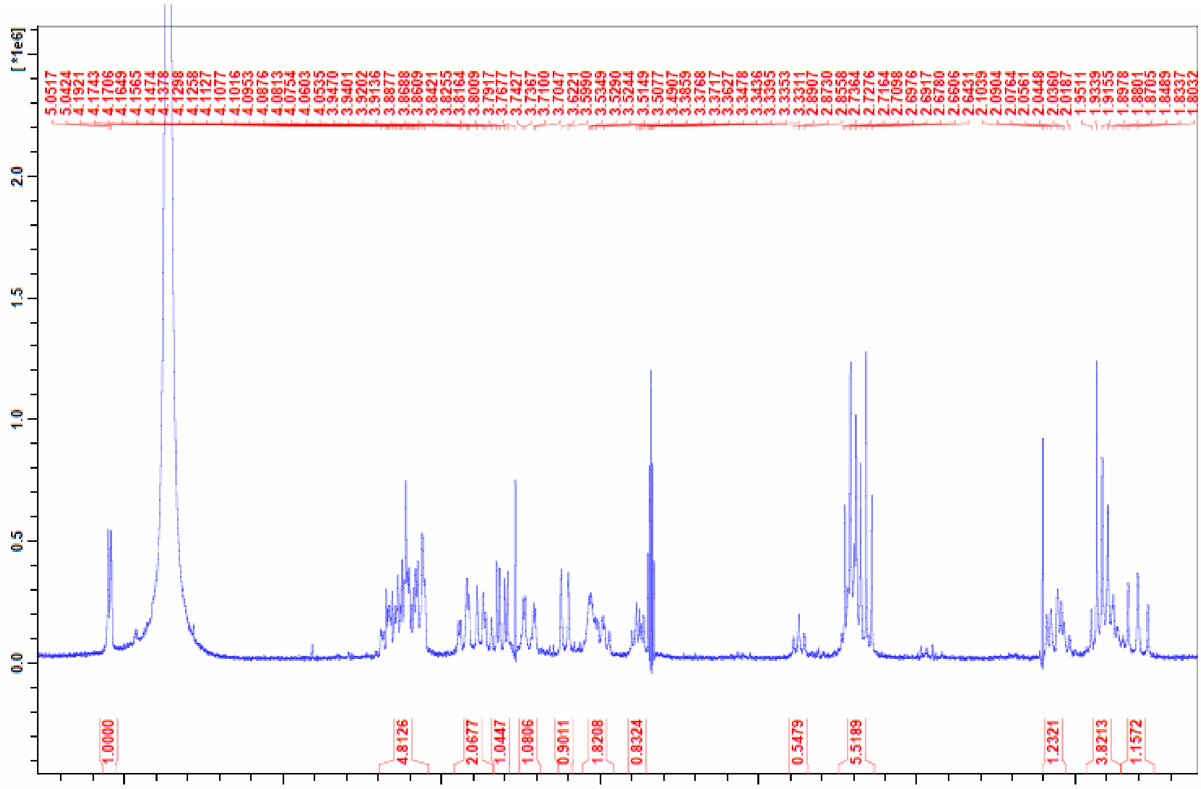

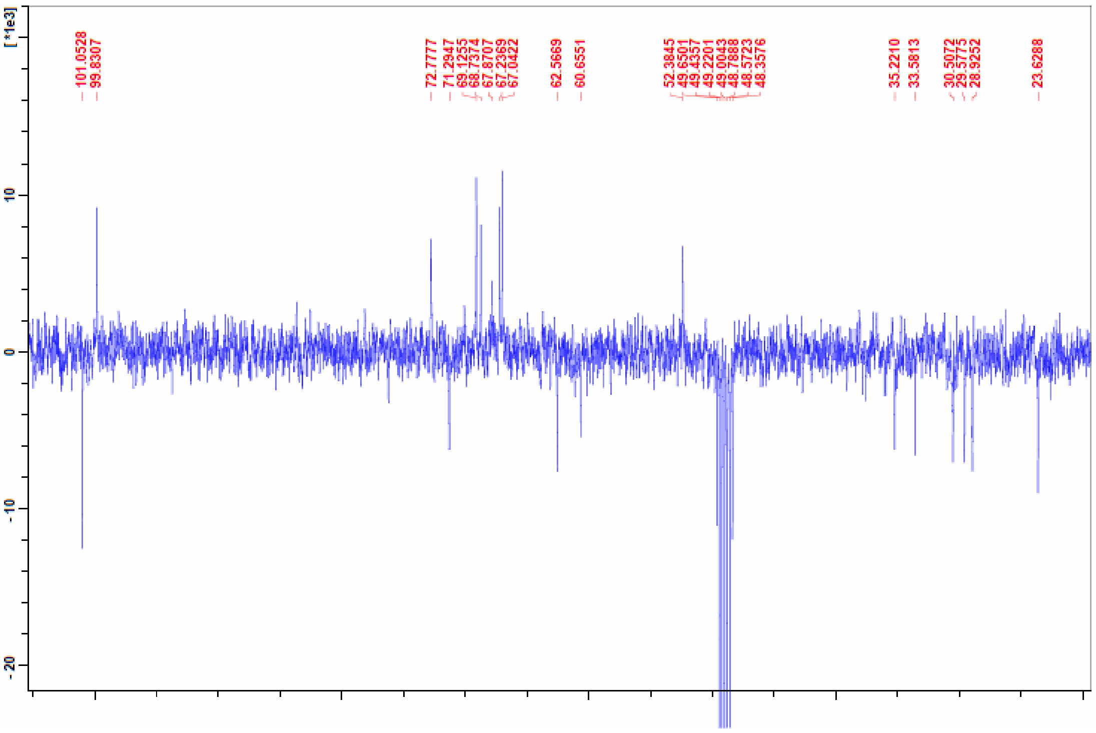

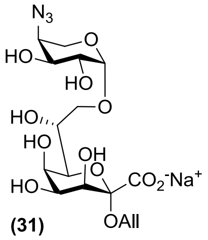

N3
HO
O
HO
O
HO
HO
OH
O
CO2-Na+
HO
OAll

(31)

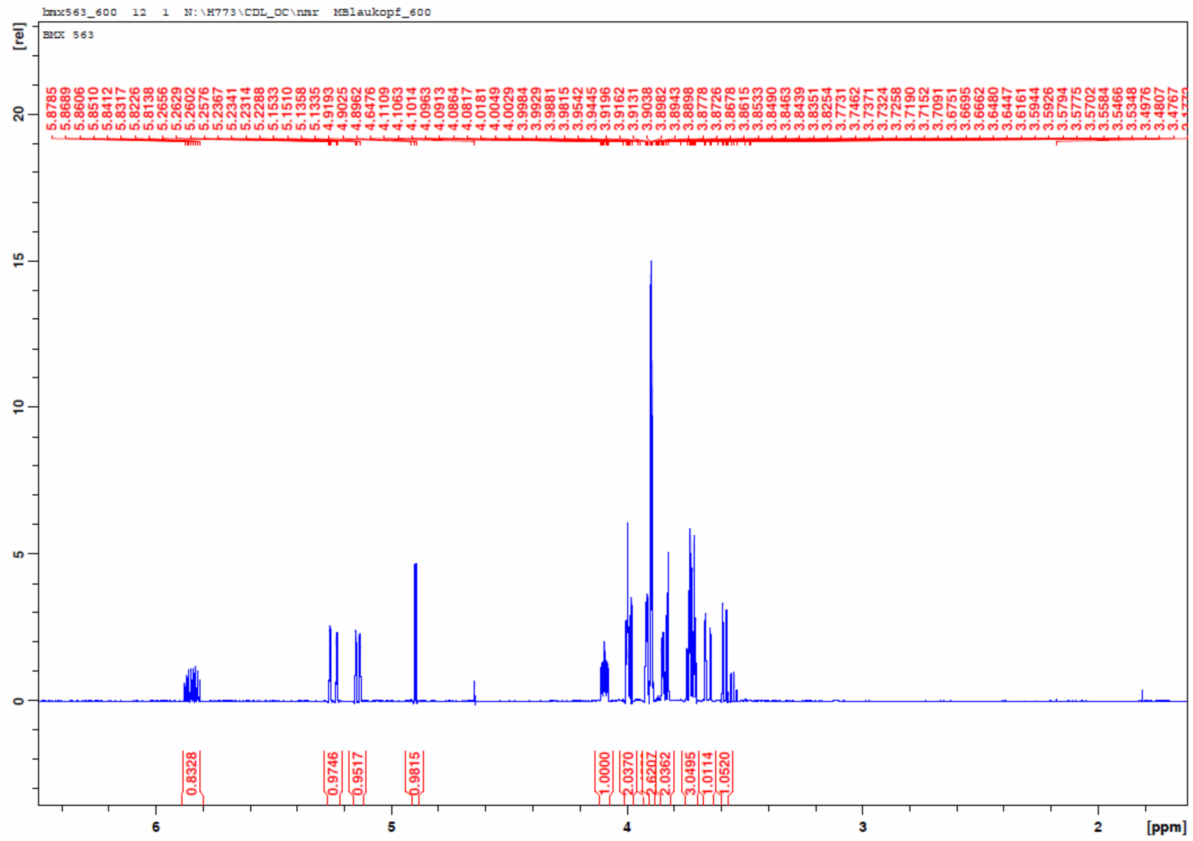

bmx563_600 12 1 N:\H773\CDL_OC\nmr MBlaukopf_600
BMX 563
[rel]
5.8785
5.8689
5.8606
5.8412
5.8317
5.8226
5.8138
5.2656
5.2629
5.2602
5.2576
5.2367
5.2341
5.2314
5.2288
5.1533
5.1510
5.1358
5.1335
4.9193
4.9025
4.8962
4.6476
4.1109
4.1063
4.1014
4.0963
4.0913
4.0864
4.0817
4.0181
4.0049
4.0029
3.9984
3.9929
3.9881
3.9815
3.9542
3.9445
3.9196
3.9162
3.9131
3.9038
3.8982
3.8943
3.8898
3.8778
3.8726
3.8678
3.8615
3.8533
3.8490
3.8463
3.8439
3.8351
3.8254
3.7731
3.7462
3.7371
3.7324
3.7258
3.7190
3.7152
3.7091
3.6751
3.6695
3.6662
3.6480
3.6447
3.6161
3.5944
3.5926
3.5794
3.5775
3.5702
3.5584
3.5466
3.5348
3.4976
3.4807
3.4767
0.8328
0.9746
0.9517
0.9815
1.0000
2.0370
2.6207
2.0362
3.0495
1.0114
1.0520
[ppm]

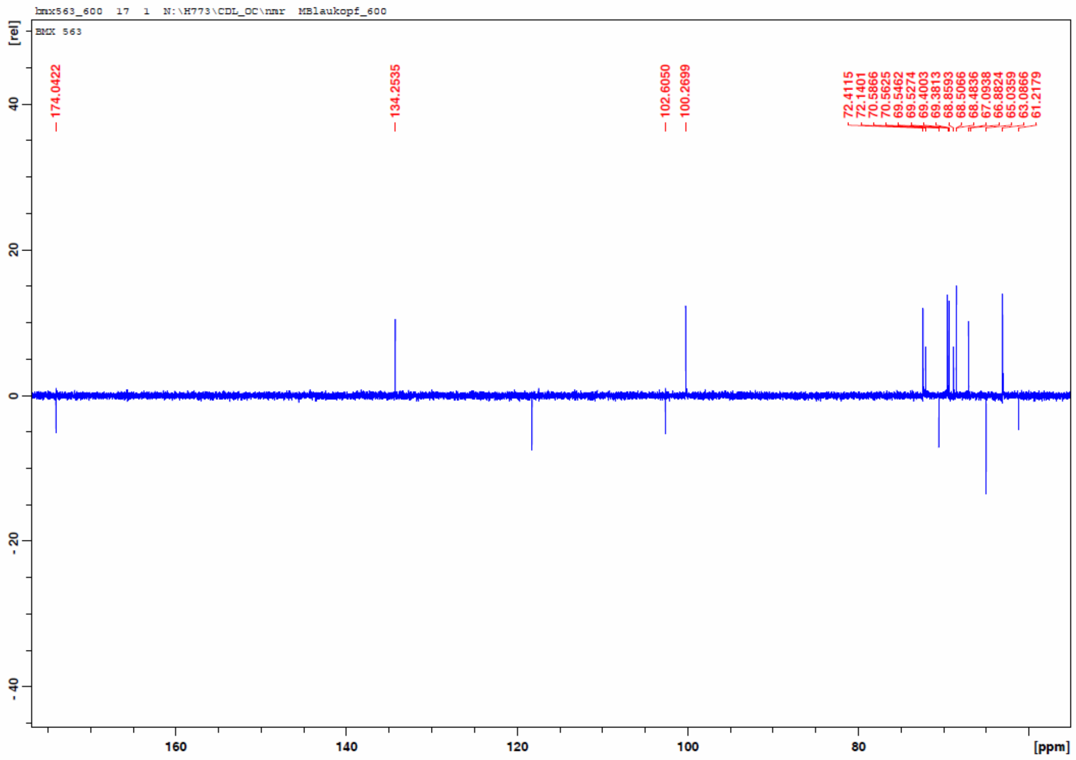

bmx563_600 17 1 N:\H773\CDL_OC\nmr MBlaukopf_600
BMX 563
[rel]
174.0422
134.2535
102.6050
100.2699
72.4115
72.1401
70.5866
70.5625
69.5462
69.5274
69.4003
69.3813
68.8593
68.5066
68.4836
67.0938
66.8824
65.0359
63.0866
61.2179
[ppm]

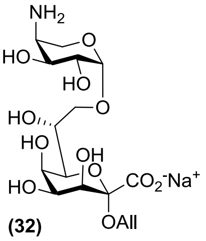

(32)

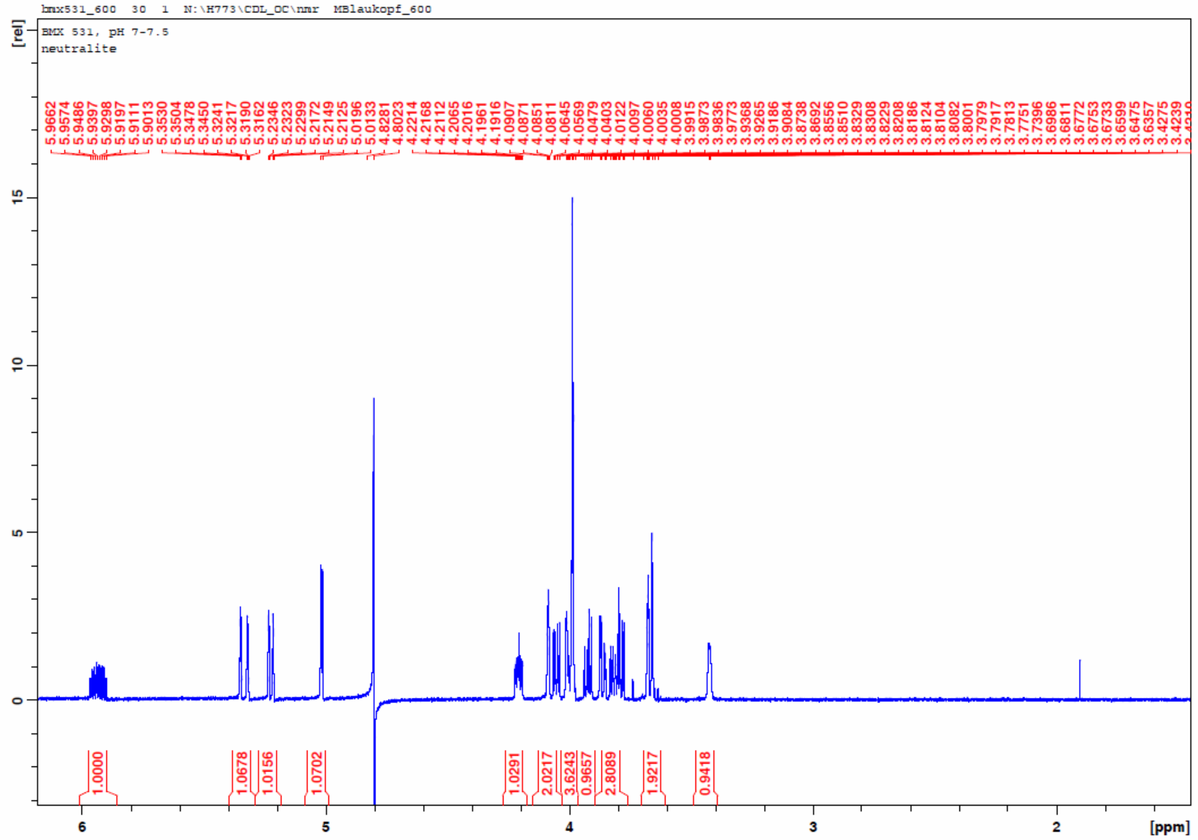

bmx531_600 30 1 N:\H773\CDL_OC\nmr MBlaukopf_600
BMX 531, pH 7-7.5
neutralite
[rel]
[ppm]

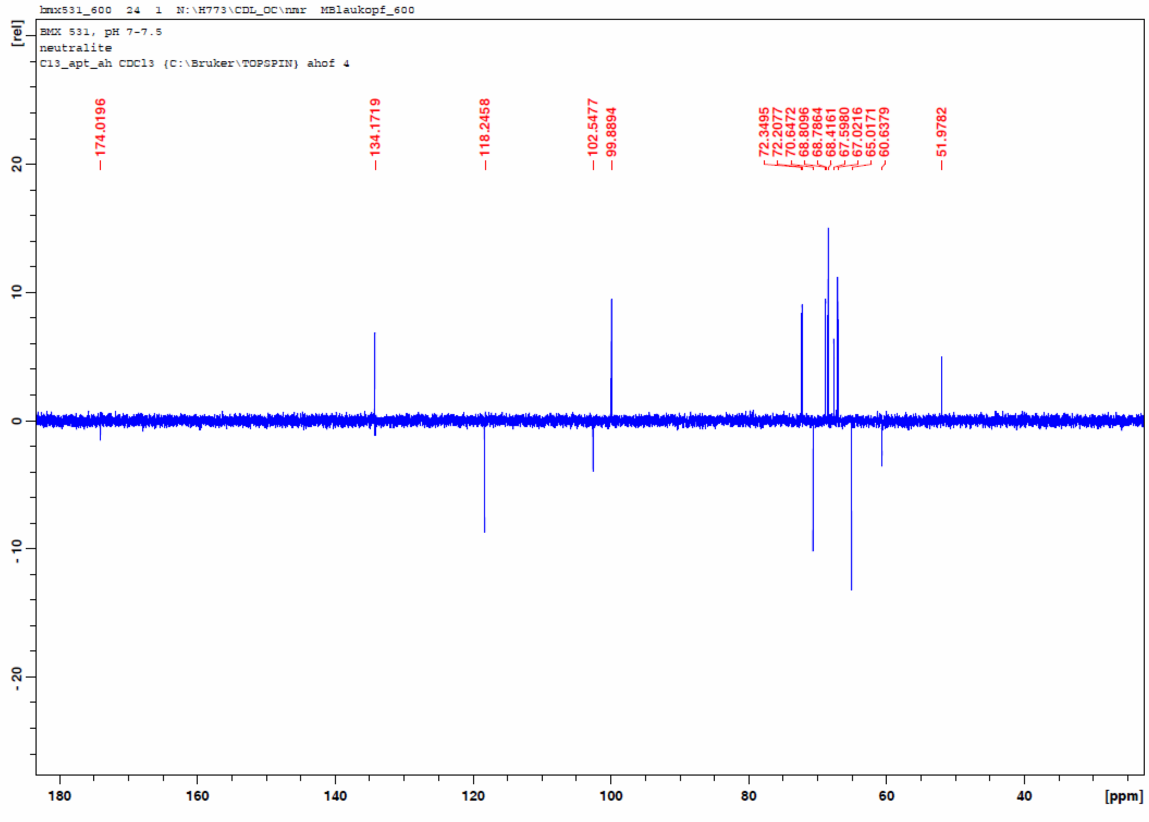

bmx531_600 24 1 N:\H773\CDL_OC\nmr MBlaukopf_600
BMX 531, pH 7-7.5
neutralite
C13_apt_ah CDCl3 (C:\Bruker\TOPSPIN) ahof 4
174.0196
134.1719
118.2458
102.5477
99.8894
72.3495
72.2077
70.6472
68.8096
68.7864
68.4161
67.5980
67.0216
65.0171
60.6379
51.9782
[rel]
[ppm]

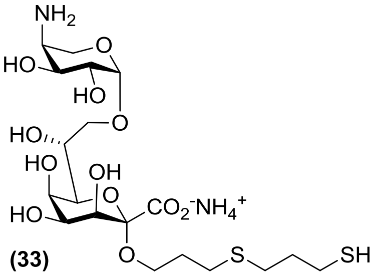

(33)

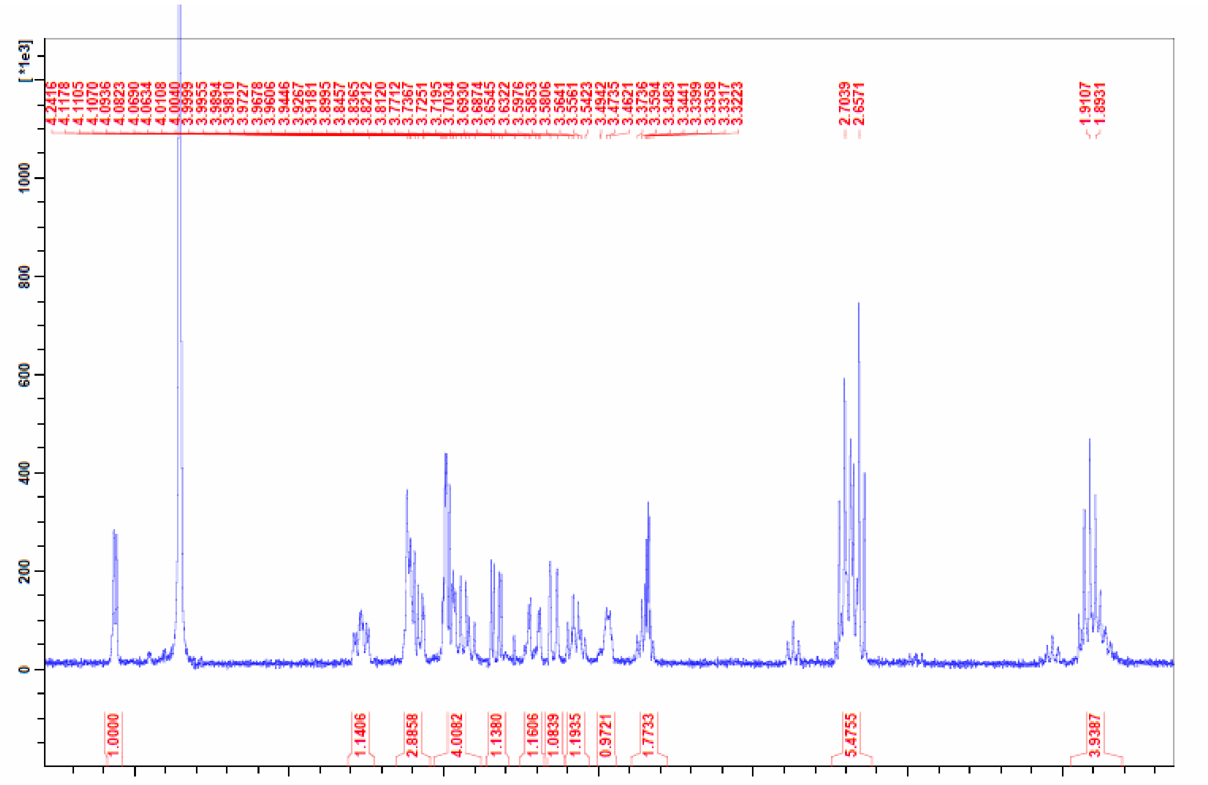

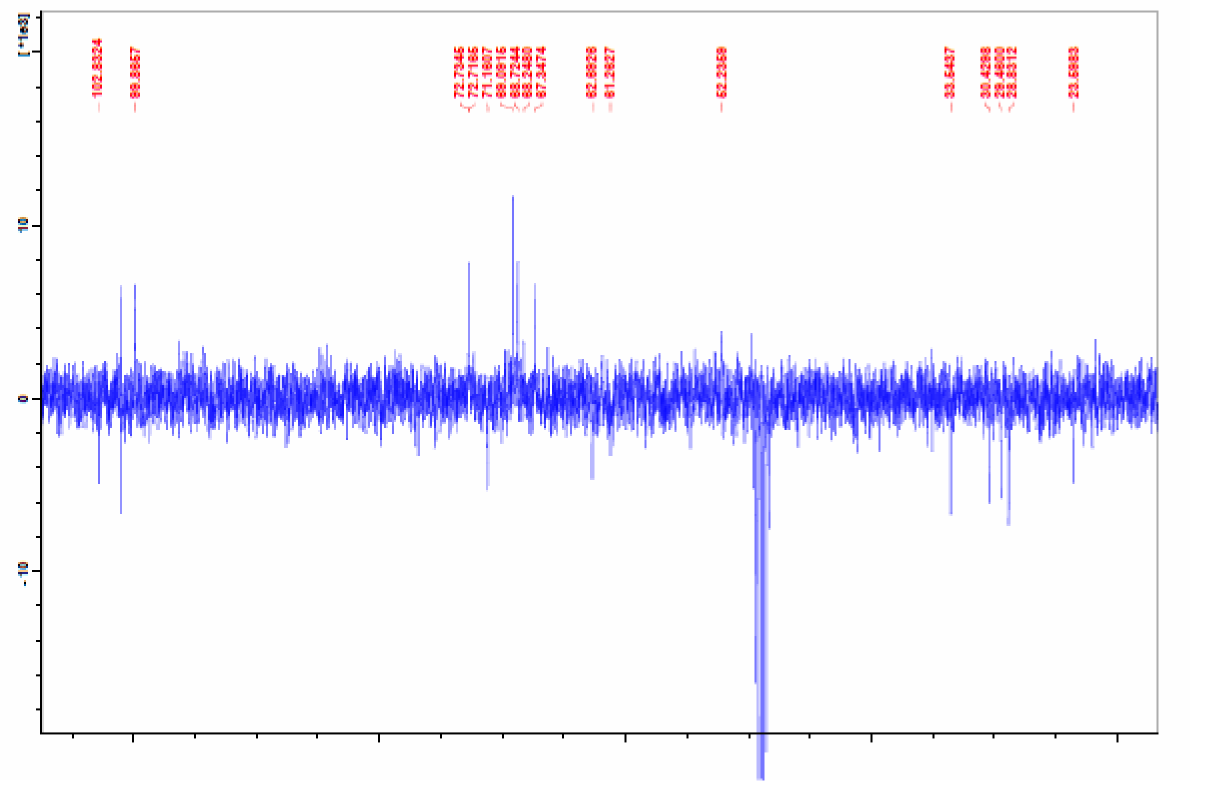

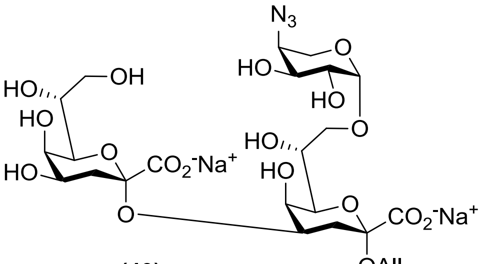

N3
HO
O
HO
O
HO
OH
HO
HO
HO
O
CO2-Na+
O
HO
HO
O
CO2-Na+
OAll

(40)

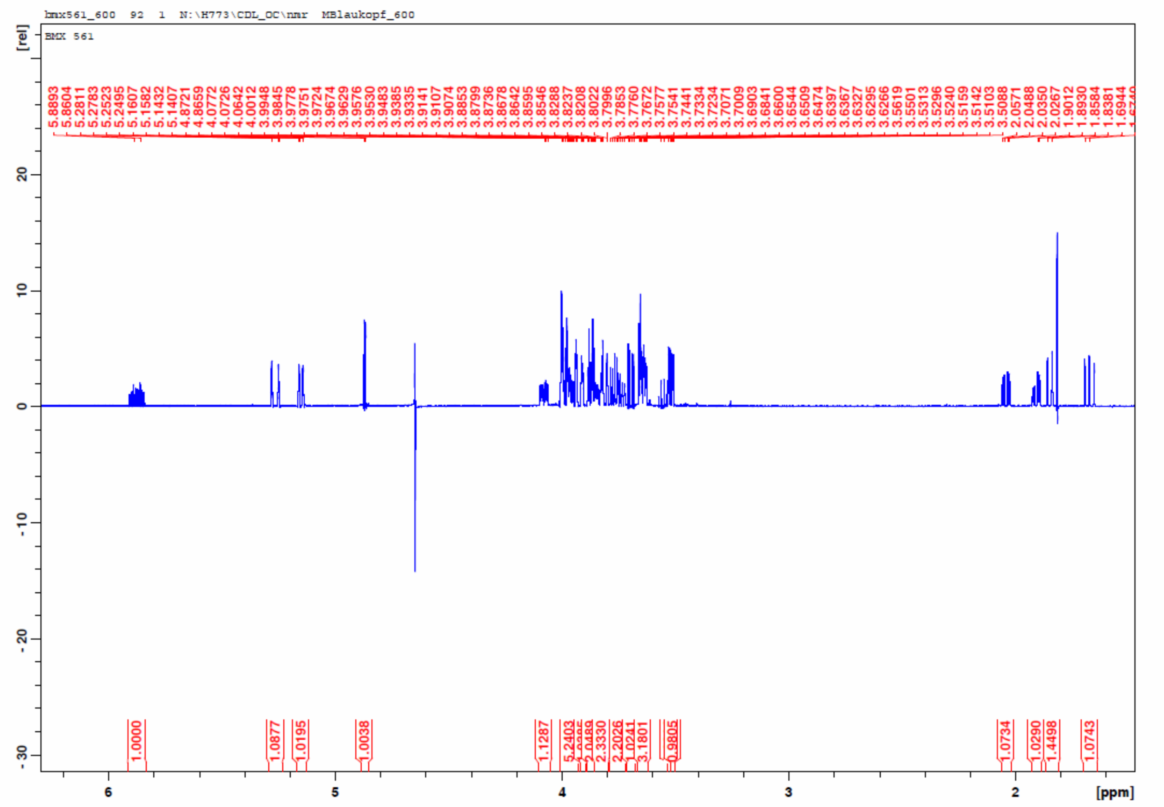

bmx561_600 92 1 N:\H773\CDL_OC\nmr MBlaukopf_600
BMX 561
[rel]
5.8893 5.8604 5.2811 5.2783 5.2523 5.2495 5.1607 5.1582 5.1432 5.1407 4.8721 4.8659 4.0772 4.0642 4.0012 3.9948 3.9845 3.9778 3.9751 3.9724 3.9674 3.9629 3.9576 3.9530 3.9483 3.9385 3.9335 3.9141 3.9107 3.9074 3.8853 3.8799 3.8736 3.8678 3.8642 3.8595 3.8546 3.8288 3.8237 3.8208 3.8022 3.7996 3.7853 3.7760 3.7672 3.7577 3.7541 3.7441 3.7334 3.7234 3.7071 3.7003 3.6903 3.6841 3.6600 3.6544 3.6509 3.6474 3.6397 3.6367 3.6327 3.6295 3.6266 3.5619 3.5501 3.5313 3.5296 3.5240 3.5159 3.5142 3.5103 3.5088 2.0571 2.0488 2.0350 2.0267 1.9012 1.8930 1.8584 1.8381 1.6944
1.0000 1.0877 1.0195 1.0038 1.1287 5.2403 2.0448 2.3330 2.2026 1.0241 3.1801 0.9805 1.0734 1.0290 1.4498 1.0743
[ppm]

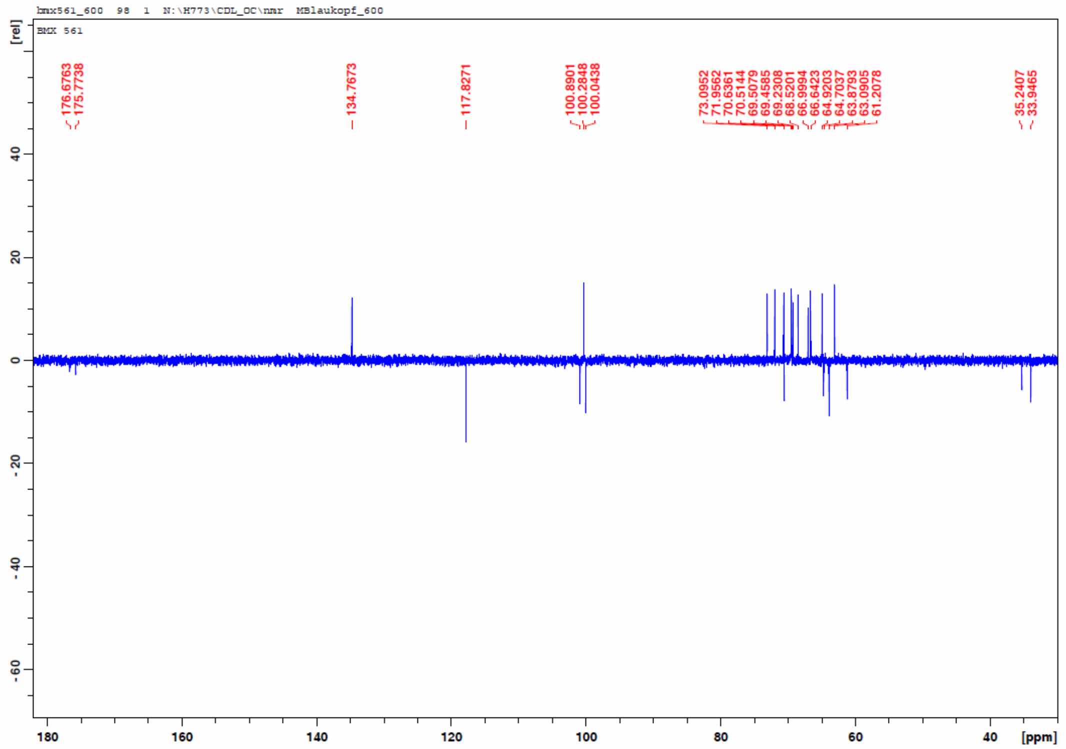

bmx561_600 98 1 N:\H773\CDL_OC\nmr MBlaukopf_600
BMX 561
[rel]
176.6763 175.7738 134.7673 117.8271 100.8901 100.2848 100.0438 73.0952 71.9562 70.6361 70.5144 69.5079 69.4585 69.2308 68.5201 66.9994 66.6423 64.9203 64.7037 63.8793 63.0905 61.2078 35.2407 33.9465
[ppm]

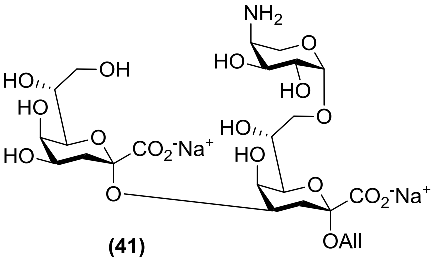

(41)

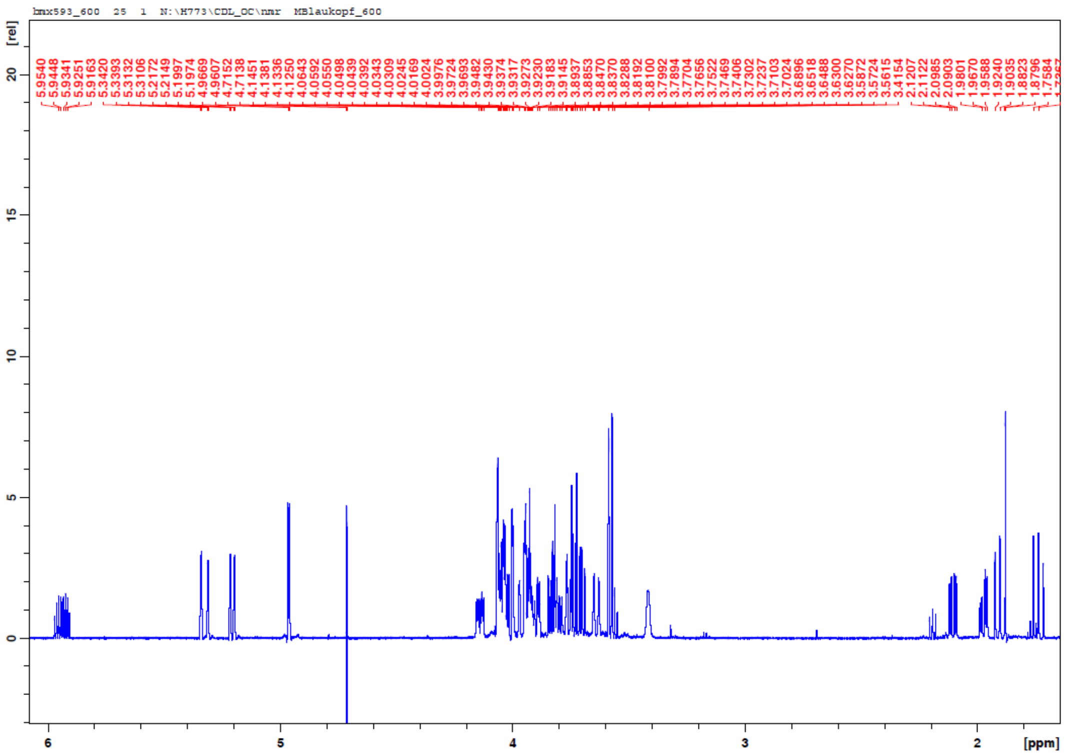

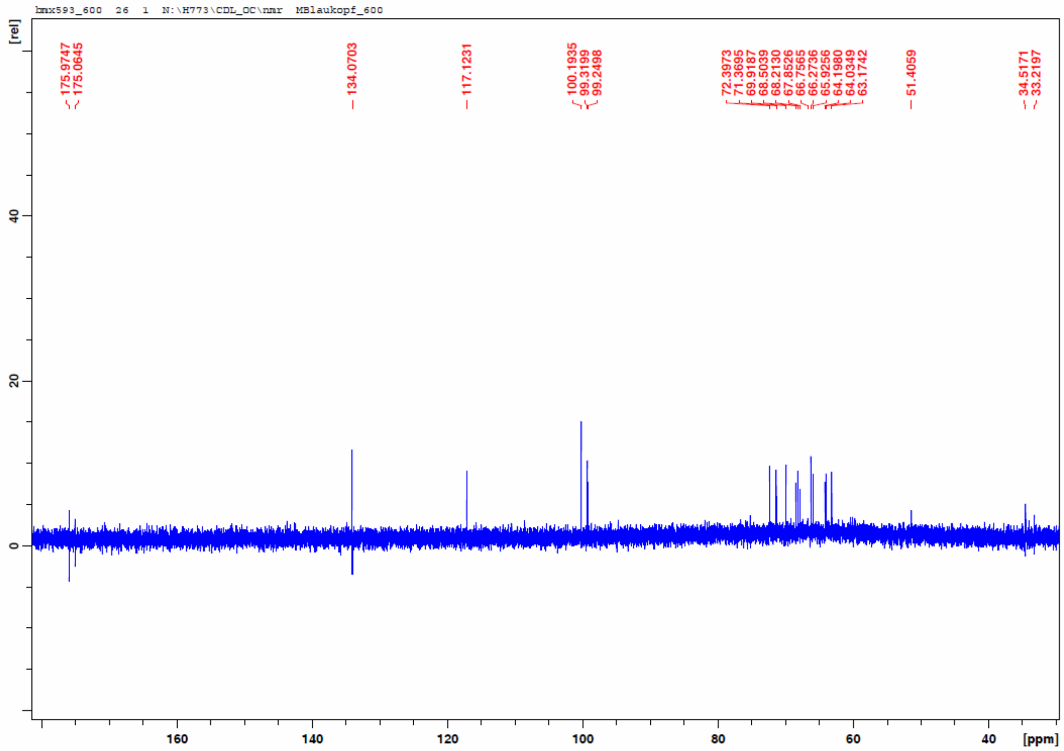

Supplement: Supplementary file 1 [file ejoc2012-0119-SD1.pdf]
